# Psychological Processes in Adapting to Dementia: Illness Representations Among the IDEAL Cohort

**DOI:** 10.1037/pag0000650

**Published:** 2021-12-09

**Authors:** Linda Clare, Laura D. Gamble, Anthony Martyr, Catherine Quinn, Rachael Litherland, Robin G. Morris, Ian R. Jones, Fiona E. Matthews

**Affiliations:** 1 REACH: The Centre for Research in Ageing and Cognitive Health, College of Medicine and Health, St Luke’s Campus, University of Exeter; 2 NIHR Applied Research Collaboration South-West Peninsula, Exeter, United Kingdom; 3 Population Health Sciences Institute, Newcastle University; 4 Faculty of Health Studies, Centre for Applied Dementia Studies, Bradford University; 5 Wolfson Centre for Applied Health Research, Bradford, United Kingdom; 6 Innovations in Dementia CIC, Exeter, United Kingdom; 7 Institute of Psychiatry, Psychology and Neuroscience, King’s College London; 8 Wales Institute for Social and Economic Research and Data, Cardiff University

**Keywords:** Alzheimer’s disease, coping, dementia representations, quality of life, well-being

## Abstract

How people understand and adapt to living with dementia may influence well-being. Leventhal’s Common Sense Model (CSM) of Self-Regulation provides a theoretical basis for exploring this process. We used cross-sectional and longitudinal data from 1,109 people with mild-to-moderate dementia in the Improving the experience of Dementia and Enhancing Active Life (IDEAL) cohort. We elicited dementia representations (DRs) using the Representations and Adjustment to Dementia Index (RADIX), a validated measure based on the CSM, identified groups sharing distinct DR profiles, and explored predictors of group membership and associations with well-being, and whether problem-focused coping played a mediating role in these associations. We identified four DR classes: people who see the condition as a disease and adopt a diagnostic label; people who see the condition as a disease but refer to symptoms rather than a diagnostic label; those who see the condition as part of aging; and those who are unsure how to make sense of the condition. A fifth group did not acknowledge any difficulties. “Disease” representations were associated with better cognition and younger age, while “aging” and “no problem” representations were associated with better mood and well-being. The association with well-being remained stable over 24 months. There was limited partial support for a mediating role of problem-focused coping. Variations in DRs may reflect individual differences in the psychological processes involved in adjusting to dementia. DRs provide a framework for personalizing and tailoring both communications about dementia and interventions aimed at supporting people in coping with dementia. There is a need to debate what constitutes a positive DR and how its development might be encouraged.

Adjusting to decline in health in later life is an adaptive task that has to be negotiated by a substantial proportion of the older population (Jaul & Barron, 2017), and the way in which this adjustment process is navigated influences well-being (Thomése & Broese van Groenou, 2006). One of the major health challenges associated with aging is the presence of dementia. Dementia affects over 50 million people worldwide and this number is predicted to increase to 152 million by 2050 (Alzheimer’s Disease International, 2019). There are currently over 5.7 million people living with dementia in the USA (Alzheimer’s Association, 2018) and over 850,000 in the UK (Prince et al., 2014). The way in which people make sense of the changes they are experiencing and adapt to living with dementia may be an important factor influencing outcomes such as well-being and quality of life (QoL), yet relatively little is known about how people diagnosed with dementia understand and adjust to their condition. Theoretical models from health psychology may help to provide insights into this process and identify better ways of supporting adjustment and promoting well-being.

The Common Sense Model (CSM) of Self-Regulation is an evolving social cognitive approach that describes the dynamic processes through which people perceive, interpret, and respond to health threats and illness-related information, and the way in which these processes influence physical, functional, and psychological outcomes independent of pathological markers of illness (Hagger et al., 2017; Hagger & Orbell, 2021; Leventhal et al., 2016). The model proposes that people develop beliefs about health and illness in general, and beliefs about specific illnesses, based on their own experience and usual functioning, experiences of those close to them, observation of others, and information from social media. These beliefs, termed prototypes, constitute a form of memory structure or schema, operating in the context of the wider self-system and sociocultural milieu. On perception of a health threat, such as a new symptom, relevant prototypes are activated and in turn generate both a representation, or mental model, of the threat, termed a cognitive illness representation (IR), and a set of emotional responses to the perceived threat (Leventhal et al., 1992, 2011, 2016). Activation of an IR and the associated emotional reactions leads to attempts at coping; for example, a belief that the health problem can be managed through lifestyle alterations might lead to changes in behavior, while a belief that nothing can be done might lead to denial or avoidance. Coping responses are not always consciously selected and may sometimes be automatic (Leventhal et al., 2016; Lowe & Norman, 2017). In the CSM, coping responses are described as reflecting “common sense” not because they are effective—they may or may not be adaptive—but because they derive from individuals’ own perceptions of the threat, rather than aligning with what would be indicated under an “expert” model (Hagger et al., 2017). Various factors can contribute to a possible mismatch between appraisal of a health threat and efficacy of the resulting coping responses, including personality, emotional reactions, and belief in one’s ability to cope, as well as beliefs about the illness or symptom itself (Hagger et al., 2017). For health providers, understanding a person’s illness-related beliefs in the context of these potential moderating factors can help to engage the person in planning how to manage the condition (Rivera et al., 2020).

IRs are commonly assessed using the Illness Perceptions Questionnaire (Moss-Morris et al., 2002), although this may not fully capture the dynamic nature of the CSM (Phillips et al., 2017). Several meta-analytic reviews provide evidence that individual elements of the IR are associated with outcomes, albeit with small-to-moderate effect sizes, across a range of physical health conditions (Dempster et al., 2015; Hagger et al., 2017; Hagger & Orbell, 2003) and in specific conditions such as stroke (Pai et al., 2019), cancer (Richardson et al., 2017), and diabetes (Hudson et al., 2014). Meta-analytic path analyses (Hagger et al., 2017) indicate that discrete elements of IRs have both direct effects on outcomes and indirect effects mediated by the types of coping strategy adopted. Representation dimensions involving higher degrees of perceived control and a more coherent understanding predict positive outcomes such as better well-being and less distress, while representation dimensions such as the identity of the condition (i.e., what it is called or how people describe it) and consequences that involve higher degrees of threat predict unfavorable outcomes such as poor well-being. However, this effect is partially mediated by coping strategy selection; avoidant coping leads to negative outcomes, but outcomes are more positive where a problem-focused coping style is adopted (Hagger et al., 2017; Hagger & Orbell, 2021). While discrete elements of the IR have often been considered separately, an alternative approach focuses on the overall IR profile and identifies groups with distinct profiles. A systematic review of studies using cluster analysis techniques to identify and compare groups with different IR profiles (Rivera et al., 2020) described clusters associated with better or worse outcomes across a number of health conditions.

A model based on perception of threat is highly relevant in the case of dementia, an umbrella term for a feared, stigmatized and ultimately terminal range of age-associated conditions with no effective disease-modifying treatment or cure. This is reflected in what is known about the dementia representation (DR) prototypes held by the general public. A survey of nearly 70,000 people in 150 countries demonstrated that most adults think they could develop dementia at some time in the future (95%), and the majority (78%) are worried about this (Alzheimer’s Disease International, 2019). In the UK, 42% of adults surveyed, and 51% of over 65s, say dementia is the health condition they are most afraid of developing (Alzheimer’s Research UK, n.d.).

The majority of the general public view dementia as part of the aging process (Alzheimer’s Disease International, 2019; Alzheimer’s Research UK, n.d.; Cations et al., 2018). However, there are also perceived associations with mental illness and the associated stigma; in the UK, half of adults surveyed thought that if they were diagnosed with dementia they would be seen by others as “mad” (Alzheimer’s Society, n.d.). In some cultures, alongside associations with aging and mental illness, symptoms of dementia may be ascribed to life challenges, bad luck, divine will or witchcraft, or explained in terms of traditional cultural and spiritual beliefs (Johnston et al., 2020). It is widely understood that there is no cure available, and while there is increasing public awareness that action can be taken to support the well-being and health of people with dementia (Cations et al., 2018), relatively few people around the world believe that adequate community services are available to make this a reality (Alzheimer’s Disease International, 2019).

Developing dementia has been conceptualized as a potential threat, not just to health, but to a person’s whole identity (Sabat & Harré, 1992). In the UK, around two-thirds of adults surveyed thought that they would no longer be the same person if they developed dementia (68%), while 62% believed that getting a dementia diagnosis would mean their life was over (Alzheimer’s Society, n.d.). In light of what is known about prototype representations of dementia, it is likely that the mental representations triggered by developing symptoms of dementia, receiving a diagnosis of dementia, or confronting the daily challenges of living with mild-to-moderate dementia would reflect high levels of perceived threat, with an associated risk of avoidant or other maladaptive coping strategies and poor psychological outcomes.

People confronted by a diagnosis of dementia and its implications adjust and cope in different ways (Bjørkløf et al., 2019; Clare, 2002, 2003), and psychological factors are strongly linked to QoL and capability to “live well” (Institute of Medicine, 2012) with the condition among people living with mild-to-moderate dementia (Clare et al., 2019; Lamont et al., 2020; Martyr et al., 2018), yet relatively little is known about the processes through which adjustment occurs and how these influence outcomes. The CSM may provide a means of clarifying the nature of the psychological processes of adjustment and the pathways that lead to better or poorer outcomes. Some initial evidence in this area has been reported. A recent scoping review (Shinan-Altman & Werner, 2019) identified six articles using the CSM as a framework for examining IRs held by people with a diagnosis of dementia. The first two qualitative studies of IRs conducted with people with dementia (Clare et al., 2006; Harman & Clare, 2006) described participants’ perceptions of the condition using the components of the CSM, suggesting that particular types of representation could impact on sense of self, psychological health, and everyday experience. For example, in the Clare et al.’s (2006) study, participants who thought nothing could be done to control the effects of the condition reported more depressive symptoms, while in the Harman and Clare’s (2006) study, participants who understood their difficulties as resulting from a progressive neurodegenerative disease spoke of how this conflicted with their wish to retain their personhood and relationships with others and created dilemmas in their daily lives, indicating that they perceived high levels of threat. These early findings were supported by a qualitative study focused on cause and control beliefs (Matchwick et al., 2014) and a qualitative case study (Glidewell et al., 2011). In a larger mixed-methods study, IRs were elicited in semistructured interviews with 64 people with dementia (Clare et al., 2016). Cluster analysis based on perceptions of identity and cause identified three clusters, termed “illness,” “aging,” and “no problem.” Compared to those who saw their difficulties as part of aging (37%), participants who saw themselves as living with an illness (45%) had better cognitive test scores and more accurate appraisals of their own functioning, but perceived more practical consequences and had lower mood. Practical consequences included finding it harder to do things, experiencing more restrictions in daily life, having less social contact, and encountering negative reactions from others. This study also provided evidence about ways of coping, with differences between clusters seen in use of problem-focused but not emotion-focused coping strategies.

The findings from Clare et al. (2016) provided the basis for developing the Representations and Adjustment to Dementia Index (RADIX), a structured interview eliciting perceptions of identity, cause, timeline, control, and practical and emotional consequences, which was validated with data from 385 participants in the Improving the experience of Dementia and Enhancing Active Life (IDEAL) cohort (Quinn et al., 2018). Applying the CSM to dementia raises some specific issues that indicate the need for a dementia-specific approach to measuring IRs. First, dementia is not a single condition but the end pathway for a number of different conditions leading to major neurocognitive disorder, and in the mild-to-moderate stages, symptom profiles differ considerably among, and even within, these conditions. Therefore, the measure must be sufficiently flexible to allow for this variability. Second, communication of the diagnosis to the person with dementia varies and may sometimes be unclear, euphemistic, or lacking altogether (Lecouturier et al., 2008; Yates et al., 2021), and a small proportion of people may not demonstrate any awareness of the diagnosis or of difficulties arising from the condition (Clare et al., 2011). It would be inappropriate to inform people of their dementia diagnosis indirectly by asking about their perceptions of the condition or to elicit representations of a condition that people do not acknowledge. The RADIX begins with a set of screening questions designed to identify people who do not acknowledge any difficulties; these individuals are not asked to complete the rest of the interview. The interview itself does not introduce specific terminology but instead allows the interviewer to use the terminology chosen by the respondent to describe the condition or the difficulties experienced. Acknowledging the need for a dementia-specific approach and the observation that many people with dementia do not conceptualize the condition in terms of illness or disease, it has been suggested that the term “dementia representations” (DRs) be used instead of referring to IRs when applying the CSM to people with dementia (Clare et al., 2016).

To summarize, the CSM describes the psychological and behavioral processes through which people conceptualize and respond to perceived threats to health, and how these processes influence coping strategies and outcomes; IRs have both direct effects on outcomes and indirect effects mediated by coping. This model may help illuminate the processes through which people with dementia adjust to and cope with the condition, the factors that influence these processes, and the ways in which processes of adjustment lead to different outcomes, including differences in capability to “live well” (Institute of Medicine, 2012) with the condition. Availability of the RADIX, a validated, dementia-specific measure, provides a structured means of eliciting DRs. This makes it possible to obtain further robust evidence by examining DRs and their associations systematically in a large sample of people with dementia. In the present study, we aimed to achieve this using data from the IDEAL cohort (Clare et al., 2014, 2019; Silarova et al., 2018). Specific objectives were as follows:

1 To describe the DRs held by people living with mild-to-moderate dementia.
2 To identify groups sharing distinct DR profiles and explore between-group differences and predictors of group membership.
3 To model the cross-sectional and longitudinal relationships between DRs and capability to “live well” with dementia.

We expected that analyses would identify groups holding different DR profiles, including “illness” and “aging” profiles as well as those who perceive “no problem,” and we hypothesized that:

1 Those holding illness-type representations would score lower on measures of “living well” than other groups, and this pattern would remain evident over time.
2 There would be between-group differences in the types of coping strategy adopted.

## Method

### Design

We used cross-sectional data from the initial assessment wave (Time 1, T1; data set version 4.5) of the IDEAL cohort (Clare et al., 2014; Silarova et al., 2018) and longitudinal data from two follow-up waves collected 1 (Time 2, T2; data set version 1.5) and 2 (Time 3, T3; data set version 1.5) years later. IDEAL was approved by the Wales Research Ethics Committee 5 (reference 13/WA/0405) and the Ethics Committee of the School of Psychology, Bangor University (reference 2014 11684). IDEAL is registered with the UK Clinical Research Network (UKCRN), number 16593.

### Participants

People living in the community in Great Britain (i.e., in England, Scotland, or Wales) with a clinical diagnosis of mild-to-moderate dementia, of any type, and a Mini-Mental State Examination (MMSE; Folstein et al., 1975) score of 15 or above were recruited to the IDEAL cohort between July 2014 and August 2016. Participants were recruited through the clinical research networks (CRNs) embedded in the UK National Health Service (NHS). CRN staff, mainly research nurses, in each of 29 participating NHS sites throughout England, Scotland, and Wales screened records and appointment lists of memory clinics and other specialist services to identify those meeting study criteria, approached potential participants either in person or in writing to provide information, invite participation, and check eligibility, and made a home visit to answer questions about the study and obtain consent. The study was also advertised on the National Institute for Health Research online Join Dementia Research portal, allowing potential participants to make contact with their local CRN team if they had not been approached directly. People who were unable to provide informed consent, who had another terminal illness, or whose home circumstances were deemed to pose a risk to visiting researchers were excluded. Those recruited were invited to nominate a family member or close friend (hereafter “caregiver”) to take part alongside them if they so wished and the caregiver agreed, but involvement of a caregiver was not mandatory. All participants with dementia and participating caregivers provided written informed consent.

T1 data were collected over a 24-month period from 2014 to 2016, T2 data were collected from 2015 to 2017, and T3 data from 2017 to 2018. The cohort comprised 1,537 people with dementia and 1,277 caregivers at T1, 1,183 people with dementia and 988 caregivers at T2, and 851 people with dementia and 759 caregivers at T3. Reasons for attrition are summarized in Table S1. Participants were visited at home on three occasions at T1 and on two occasions each at T2 and T3 to complete the assessment. People with dementia completed their questionnaires in an interview with a researcher while caregivers concurrently completed their questionnaires independently, with support from the researcher if required.

### Measure

#### Demographic Characteristics

Demographic characteristics considered for participants with dementia were as follows: age; sex; ethnicity (White British, other); educational qualifications (no qualifications, school leaving certificate at age 16, school leaving certificate at age 18, university); socioeconomic status, classified as I/II (professional/managerial and technical), III-NM/III-M (skilled nonmanual/skilled manual), IV/V/armed forces (partly skilled/unskilled), not applicable/missing, as derived from UK Office for National Statistics occupational classifications (Office for National Statistics, 2010); living situation (lives alone, lives with spouse/partner, other living arrangement, as outlined in Clare et al., 2020); dementia diagnosis, classified as Alzheimer’s disease (AD), vascular dementia (VaD), mixed AD/VaD, frontotemporal dementia (FTD), Parkinson’s disease dementia (PDD), dementia with Lewy bodies (DLB), and other/unspecified (these were simplified to AD/VaD/Mixed AD/VaD vs. Other for the multinomial regression); and time elapsed since diagnosis (<1 year, 1–2 years, 3–5 years, 6+ years from baseline assessment). Where a caregiver participated in the study alongside the person with dementia, the relationship of the caregiver to the person with dementia was noted (spouse/partner, other family member/friend).

#### Measures Completed by Participants With Dementia

DRs were assessed with the RADIX (Quinn et al., 2018). An initial set of screening questions identifies whether or not the respondent acknowledges any problems or difficulties indicative of dementia. The full measure is administered only to those who score positively on screening. The first question asks what the person calls the condition or difficulties acknowledged in response to the screening questions, and this term is used by the interviewer in the questions that follow. The questions examine perceptions of identity, cause, timeline, potential for control, and consequences, as follows: identity (what the person calls the condition, categorized into diagnostic label, label describing symptoms, label describing emotional reactions, don’t know, other) with a follow-up question aimed at eliciting awareness of the diagnosis if not spontaneously mentioned (“what does the doctor call it?”); cause (categorized into brain changes or disease, aging, lifestyle or life events, don’t know); timeline (get better, stay the same, get worse, unsure); possibility of control (strongly agree, agree, disagree, strongly disagree); experience of practical consequences (sum of scores on 4 items each rated on a 4-point Likert scale from *strongly disagree* to *strongly agree*; example item: as a result of my … I cannot do some of the things that I used to do); and experience of emotional consequences (sum of scores on 5 items each rated on a 4-point Likert scale from *strongly disagree* to *strongly agree*; example item: as a result of my … I feel I have lost confidence in myself). In the IDEAL interview, administration of the timeline item differed from the validated measure in that the possible outcomes (get better, stay the same, get worse) were presented as three separate questions rather than one single question.

Participants with dementia who completed the RADIX also responded to questions about perceived stigma and coping styles. Perceived stigma was assessed with four items from the Stigma Impact Scale (Burgener & Berger, 2008; Fife & Wright, 2000); one item was taken from each of the four subscales, and all items were rated on a 4-point Likert scale from 1 = *strongly disagree* to 4 = *strongly agree*, with higher scores indicating greater perceived stigma. The items used were as follows: I feel I have been treated with less respect than usual by others (social rejection subscale); I have experienced financial hardship that has affected how I feel about myself (financial insecurity subscale); I feel others think I am to blame for my [condition/difficulties] (internalized shame subscale); I feel set apart from others who are well (social isolation subscale). In line with the approach taken in the RADIX, the interviewer employed the term used by the participant to describe the condition or the associated difficulties (e.g., Alzheimer’s, dementia, memory problems).

Coping styles were assessed with 12 items based on the coping data elicited in the interviews which formed the basis for developing the RADIX measure (Clare et al., 2016). For all items responses were made on a 4-point Likert scale from 1 = *strongly disagree* to 4 = *strongly agree*. The set of 12 coping items was developed as follows. Fifteen items representing the range of practical and emotional coping strategies described in the interviews were identified for inclusion in the IDEAL interview at Time 1. The items were phrased in a way that was similar to the wording used by participants in the interviews and adopted the same convention as the RADIX whereby the interviewer inserted the term used by the participant to describe the condition or difficulties experienced. Items focusing on specific memory strategies were not included as, in contrast to the 2016 study where participants had a diagnosis of Alzheimer’s disease and hence memory difficulties were likely to be particularly salient, IDEAL cohort participants had various types of dementia. To align these items with typologies of coping typically used in studies of IRs, five expert raters were asked to classify the items into one of the categories identified and defined by Hagger et al. (2017, Online Supplemental Material, Appendix G): avoidance, cognitive reappraisal, emotion venting, problem-focused, and seeking social support. In a first round of rating, consensus was achieved on the classification of 10 items, and in a second round, consensus was reached on a further 3 items where one rater had differed from the rest in the first round resulting in 80% agreement. Two items that achieved less than 80% agreement in the first round were considered too ambiguous and were discarded. This process yielded a set of 13 items, classified as problem-focused (6 items), cognitive reappraisal (2 items), avoidance (4 items), and seeking social support (1 item). Next, we explored whether these groupings were supported statistically. Using a polychoric correlation matrix, the results of a Kaiser–Meyer–Olkin test (0.72) and a significant Bartlett’s test (*p* < .001) suggested that the items were suitable for factor analysis. Factor analysis using the psych package in R yielded a four-factor solution. The problem-focused and cognitive reappraisal items (6 and 2, respectively) loaded together onto a single factor. Three of the four avoidance items loaded together onto a second factor. The social support item loaded onto a third factor. A further item classified by the expert raters as avoidance was the sole item loading onto a fourth factor and was discarded. Thus the final set of 12 items comprised 6 assessing problem-focused coping (e.g., I find it helps to keep to a routine), 2 assessing cognitive reappraisal (e.g., Due to my [condition] I have to accept the changes in my life), 3 assessing avoidance (e.g., I prefer not to talk about my [difficulties]), and 1 assessing seeking social support (I rely on others for help). Sums of the variables within the four coping types were used for analysis. The coping items are shown in Table S2 with details of classification by expert raters and results of the factor analysis.

Cognition was assessed with the Addenbrooke’s Cognitive Examination-III (ACE-III; Hsieh et al., 2013); this yields a total score out of 100 with higher scores indicating better cognitive function. Comorbidity was assessed with the Charlson Comorbidity Index age-adjusted score (CCI; Charlson et al., 1987, 2008); this measure records the presence of any of 23 chronic conditions (e.g., hypertension), each of which is assigned a weighted score indicating the associated mortality risk, with the sum of scores adjusted for age. Where a caregiver was participating alongside the person with dementia, s/he was asked to support the completion of this measure by the person with dementia.

Attitudes Toward Own Aging (ATOA) were assessed with the 5-item ATOA subscale of the Philadelphia Geriatric Center Morale Scale (PGCMS; Lawton, 1975) using a pro-rata score to account for missingness when one of the five responses was missing (Sabatini et al., 2021); respondents agree or disagree with each statement (example item: Do you feel that as you get older you are less useful?), and items are scored such that a higher total score reflects more positive ATOA. Self-efficacy was assessed with the 10-item Generalized Self-Efficacy Scale (Schwarzer & Jerusalem, 1995); responses (example item: I am confident that I could deal efficiently with unexpected events) are scored on a 4-point Likert scale from 1 = *not at all true* to 4 = *completely true*, with higher scores indicating greater perceived self-efficacy. Depressive symptoms were assessed with the 10-item Geriatric Depression Scale (GDS-10; Almeida & Almeida, 1999); respondents answer yes or no to each item (example item: Do you feel pretty worthless the way you are now?) and higher scores indicate higher levels of depressive symptoms.

Capability to “live well” with dementia was assessed with three measures. The 13-item Quality of Life in Alzheimer’s Disease scale (QoL-AD; Logsdon et al., 1999, 2000) is a dementia-specific measure of QoL, with items (example item: How do you feel about your energy level?) scored on a 4-point Likert scale from 1 = *poor* to 4 = *excellent* and higher scores indicating better QoL. The 5-item Satisfaction with Life Scale (SwLS; Diener et al., 1985) elicits a global judgment about one’s life (example item: In most ways my life is close to my ideal), with responses made on a 7-point Likert scale from 1 = *strongly disagree* to 7 = *strongly agree* and higher scores indicating greater satisfaction. The 5-item World Health Organization-Five Well-Being Index (WHO-5; Bech, 2004) measures psychological well-being over the previous 2 weeks; items (example item: I have felt cheerful and in good spirits) are rated on a 6-point scale from 0 = *at no time* to 5 = *all of the time*, and ratings are summed and converted to a percentage score, with higher scores indicating better well-being.

#### Measures Based on Informant Reports From Caregivers

Caregivers provided details of number and severity of neuropsychiatric symptoms experienced by the person with dementia using the Neuropsychiatric Inventory Questionnaire (NPI-Q; Kaufer et al., 2000; Morris, 2008). This covers 12 symptom domains, including apathy, delusions, hallucinations, and agitation; caregivers indicate whether or not a given symptom is present and if so rate its severity (mild, moderate, or severe) and indicate the degree of caregiver distress that it provokes on a 6-point Likert scale from 0 = *not distressing at all* to 5 = *extremely distressing*. Caregivers rated the functional ability of the person with dementia using the 11-item version of the Functional Activities Questionnaire (FAQ; Martyr et al., 2012; Pfeffer et al., 1982); this covers 11 instrumental activities of daily living such as shopping, recording financial transactions, keeping abreast of current events, and using the telephone, and each activity is rated on a 4-point Likert scale from 0 = *normal, as s/he has always done; never did but could do now* to 3 = *dependent on others* with higher scores indicating poorer functional ability.

#### Caregiver Self-Report Measures

Caregiver stress was assessed with the 15-item Relative Stress Scale (RSS; Greene et al., 1982), a measure of perceived stress resulting from the caregiving role (example item: Do you ever feel frustrated with your relative/friend?); items are rated on a 5-point Likert scale ranging from 0 = *not at all* to 4 = *always/considerably*, with higher scores indicating greater stress. Role captivity was assessed with the 3-item Role Captivity Questionnaire (Pearlin et al., 1990); items (example item: How much do you wish you were free to lead a life of your own?) are scored on a 4-point Likert scale from 1 = *not at all* to 4 = *very much*, with higher scores indicating a greater sense of feeling trapped. Competence was assessed with the 3-item Caregiving Competence Scale (Robertson et al., 2007); items (example item: How often do you feel that you are doing a good job as a caregiver?) are rated on a 4-point Likert scale ranging from 1 = *never* to 4 = *all of the time*, with higher scores indicating greater perceived competence. The experience of rewards and satisfactions in caregiving was assessed with the 9-item Positive Aspects of Caregiving Questionnaire (Tarlow et al., 2004); items (example item: Providing help to my relative/friend has made me feel appreciated) are rated on a 5-point Likert scale from 1 = *disagree a lot* to 5 = *agree a lot*, with higher scores indicating perception of more positive aspects of caregiving.

#### Statistical Analyses

We first described the RADIX responses for all participants with dementia who completed the measure. A latent class analysis was conducted using Mplus Version 8.2 (Muthén & Muthén, 1998–2017) to identify groups based on responses to a set of observed indicators from the RADIX questionnaire: identity, cause, timeline, and potential for control. A two-class solution was fitted first, followed by iterative solutions with additional numbers of classes. The solutions were evaluated using the Bayesian information criteria (BIC), sample size adjusted BIC (ssBIC), entropy, Lo-Mendell-Rubin likelihood ratio test (LMR-LRT), and bootstrapped parametric likelihood ratio test (BLRT); LMR-LRT and BLRT compare improvement of fit between sequential class models. Missing data were handled using full information maximum likelihood (FIML) estimation.

Differences across classes in each RADIX component and measure of coping were examined using the (BCH; Bolck et al., 2004; continuous variables) or categorical distal outcome (DCAT; categorical variables) methods available in Mplus, with Holm–Bonferroni correction for multiple comparisons. Where a variable was statistically significant, post hoc comparisons were conducted and pairwise differences with an adjusted *p* value of <.008 (Bonferroni corrected to account for 6 two-tailed comparisons) were considered significant and reported. We then examined whether the four classes, plus a “no problem” group consisting of individuals who responded negatively to all screening questions, differed significantly in terms of demographic, dementia-related, and psychological factors, again using the BCH and DCAT methods, with Holm–Bonferroni correction for multiple comparisons. For measures with significant differences, post hoc comparisons were conducted and pairwise differences with an adjusted *p* value of <.005 (Bonferroni corrected to account for 10 two-tailed comparisons) were considered significant and reported. For participants with a participating caregiver, we also explored whether there were differences in caregivers’ experiences of caregiving. Multinomial regression was employed to examine predictors of group membership, using the BCH method in Mplus to account for misclassification error (Asparouhov & Muthén, 2014).

Linear growth curve modeling (LGCM) was conducted in Mplus to examine whether group membership predicted ability to “live well” with the condition both cross-sectionally at Time 1 (intercept) and longitudinally over Times 1–3 (slope). FIML estimation was used, which draws on all available data present for a given measurement occasion to estimate parameters. The measurement model is shown in Figure S1. All models had good model fit indices: normed chi-square index (χ^2^/*df*) < 3.0, comparative fix index > 0.95, Tucker–Lewis Index (TLI) > 0.95, root-mean-square error of approximation (RMSEA) < 0.08 (Hu & Bentler, 1999; Kline, 2015; Tucker & Lewis, 1973). The association of group membership with QoL, satisfaction with life, and well-being was assessed both at baseline and over time using both unadjusted models and models adjusted for age, sex, and dementia subtype. In the event that group membership predicted ability to “live well,” mediation analyses were planned to determine whether the association was mediated by coping style. Mediation analysis was conducted in Mplus using structural equation modeling with 1,000 bootstrapped confidence intervals to obtain direct and indirect effects. Models were run using both baseline data and data across time (class at Time 1, coping at Time 2, and living well measures at Time 3). In the LGCM and mediation analyses, class membership was weighted by the posterior probabilities to account for uncertainty.

## Results

Participants were the 1,109 people with dementia in the IDEAL cohort at T1 whose data were not used in the RADIX validation study. Of these, 1,033 participants (93%) endorsed at least one item on the RADIX screening checklist and went on to complete the full RADIX measure, while 76 (7%) answered “no” to all the RADIX screening questions, indicating that they perceived no difficulties (“no problem” group). Participant characteristics are summarized in Table 1.[Table tbl1]

RADIX responses covered perceived identity of the condition or difficulties, awareness of diagnosis, perceived cause, timeline or prognosis, and availability of ways of managing the effects of the condition. Regarding identity, just over a quarter of participants responded by spontaneously giving a specific diagnosis. Over half of the respondents instead used a descriptive term to characterize the difficulties; in most cases, this was related to particular symptoms, such as “memory problems,” but in some cases, descriptive terms were related to emotional responses (e.g., “It makes me annoyed. It makes me cry. It’s distressing”) or were more general in nature (e.g., “things are different”). Smaller proportions described their difficulties as aging, thought they did not have any difficulties, or said they did not know how to describe their difficulties. Those participants who did not provide a diagnostic label were asked specifically whether they were aware that a diagnosis had been made (“what does the doctor call it?”). Combining these responses with those of the participants who offered a diagnostic label without prompting, about two-thirds of the whole sample were aware of a diagnosis and just over half were able to give the diagnosis. Regarding perceived cause, the most frequently chosen responses were “don’t know,” “aging,” and “changes in the brain,” each selected by approximately a quarter of participants. Regarding timeline, just under half thought the condition would worsen over time, while a third were unsure and a few thought it would improve. The majority, about two-thirds, thought there were some possibilities for controlling the effects of the condition or difficulties. Details of responses to the RADIX and coping items for all those completing the full RADIX can be found in Table 2.[Table tbl2]

Latent class analysis based on identity, cause, timeline, and control was conducted using data from 1,033 participants with dementia. As shown in Table S3, both the three- and four-class solutions had the best fit indices; for the three-class solution, BIC = 12,548, ssBIC = 12,361, LMR-LRT *p* < .001, BLRT *p* < .001, and for the four-class solution, BIC = 12,635, ssBIC = 12,383, LMR-LRT *p* = .008, BLRT *p* < .001. BIC and ssBIC were lower for the three-class solution, but the LMR-LRT and BLRT suggested that the four-class solution was an improvement on the three-class solution. Based on these results, and on entropy and interpretability of the classes, the four-class solution was taken forward. The entropy of 0.819 suggests a high level of certainty in the classification of individuals (Celeux & Soromenho, 1996; Tein et al., 2013). Distributions of each of the four latent classes for categorical identity and cause variables, and for ordinal timeline and control variables, are shown in Table S4. Initial testing of differences across classes was done using the BCH and DCAT methods in Mplus.

Classes were characterized as follows. “Disease-diagnosis” (*n* = 141.9, 13.7%) comprised individuals who used a diagnostic label, identified the cause as related to brain changes or disease, and mostly believed the condition would get worse over time. “Disease-symptoms” (*n* = 504.4, 48.8%) comprised individuals who described the condition in terms of symptoms rather than a diagnostic label, but identified the cause as related to brain changes or disease, and again mostly believed the condition would get worse over time. “Aging” (*n* = 117.2, 11.3%) comprised individuals who mostly described the condition in terms of symptoms, attributed these to aging, and perceived fewest practical or emotional consequences. “Unclear” (*n* = 269.4, 26.1%) comprised individuals who described symptoms or in some cases a diagnosis, either did not know the cause or attributed the difficulties to brain changes, and did not believe their condition would worsen over time; the representations held by these individuals could be considered low in coherence. Significant differences between the classes were observed for all RADIX dimensions and for perceived practical and emotional consequences; results are summarized in Table 3. Responses to the coping items are also summarized in Table 3. There were significant differences among the classes for problem-focused coping, where the “aging” class had the lowest mean score, and seeking social support, where the two “disease” classes had higher mean scores than the “aging” and “unclear” classes. However, numerical differences were very small and post hoc tests showed no differences for the seeking social support method of coping when accounting for multiple comparisons. There was no significant between-class difference for cognitive reappraisal or avoidant coping.[Table tbl3]

Comparison of participants in the four classes plus the “no problem” group showed significantly different patterns in relation to age and dementia subtype, but no differences with regard to sex, socioeconomic status, or education; details can be found in Table S5a. Participants in the “disease-diagnosis” class tended to be younger. Significant differences between the “disease” classes and the “aging” and “unclear” classes were evident with regard to dementia subtype; VaD was more prevalent in the “disease-diagnosis” class while the “aging” class had fewer people overall with rarer subtypes. There was a significant difference across groups for the categorical variable of time since diagnosis, and mean time elapsed since diagnosis was highest in the “disease-diagnosis” group. There were no differences in caregiver status or living situation across the five groups.

Comparison of the five groups on dementia-related variables, measures of psychological characteristics and health, and measures of “living well” (Table S5b) showed that those in the “disease” classes were likely to have better cognition, but poorer scores for QoL, satisfaction with life, and well-being, lower self-efficacy, more negative attitudes toward their own aging, and more symptoms of depression than participants in other classes. In contrast, participants in the “aging” and “no problem” groups tended to have higher scores for QoL, satisfaction with life, well-being, and self-efficacy, more positive attitudes to aging, and fewer symptoms of depression. It should be noted, though, that absolute differences in mean scores among the groups were relatively small. There were no significant differences in functional ability, number of comorbid health conditions, or perceived stigma across the groups (stigma was not assessed in the “no problem” group). There were no significant differences in caregivers’ levels of stress, distress at symptoms, feelings of role captivity, sense of competence, or endorsement of positive aspects of caregiving across the five groups and no differences in number or severity of caregiver-reported neuropsychiatric symptoms (Table S5c).

Multinomial regression was conducted to examine associations with group membership (Table 4) using the “disease-symptoms” class as the reference category. This class was chosen because evidence from previous studies suggested that disease-type representations were associated with lower well-being compared to other types, and of the two “disease” classes identified here, the “disease-symptoms” profile was more prevalent. In the initial univariable model, shown in Table 4 (Model 1), significant associations with class membership were seen in all domains except for dementia subtype, time since diagnosis and functional ability. To check whether dementia severity impacted on the estimates, we conducted a sensitivity analysis on the univariable model incorporating as covariates two indices, MMSE score as a categorical variable and time elapsed since diagnosis; including these had minimal impact on the results, as shown in Table S6. Based on the results of the multivariable model (Table 4, Model 2), in comparison to the “disease-symptoms” class, the main differences observed were related to cognitive ability and age. With regard to cognitive ability, the “disease-diagnosis” class was similar to the “disease-symptoms” class, while all other groups were more likely to have lower cognitive test scores. With regard to age, the “disease-diagnosis” class was more likely to be younger and the “aging” class more likely to be older. In addition, compared to the “disease-symptoms” class, the “disease-diagnosis” class had higher age-adjusted comorbidity scores and the “unclear” class had higher scores for self-efficacy.[Table tbl4]

We next explored the effect of class membership on “living well” measures longitudinally. We examined the “living well” measures both at Time 1 (intercept) and as time progressed (slope), as shown in Table 5. Because calculation of the slope requires at least two data points per individual, the class compositions for those with two or more time points for QoL-AD, SwLS, and WHO-5 were examined to check that the reduced sample size still represented the classes (Table S7). Relative to the “disease-symptoms” class, mean “living well” scores at baseline were lower for the “disease-diagnosis” class, with mean decreases of 1.47 points for QoL-AD, 3.30 for SwLS, and 8.24 for WHO-5, but higher for the “aging” (mean increases of 2.50 points for QoL-AD, 2.73 for SwLS, and 8.09 for WHO-5) and “unclear” (mean increases of 1.90 points for QoL-AD, 2.19 for SwLS, and 6.25 for WHO-5) classes. The “no problem” group had the highest scores on each “living well” measure overall, with mean increases of 4.77 points for QoL-AD, 3.39 for SwLS, and 11.44 for WHO-5. Over time, there was little change in the “living well” measures. There were no significant changes in the trajectories of QoL-AD, SwLS, or WHO-5 for the “diagnosis-disease” class compared to “disease-symptoms” and no significant differences for the “aging” and “no problem” groups. For the “unclear” class, a small but significant decline was seen for SwLS and the trend was also observed for QoL-AD and WHO-5. The greatest differences were those seen at baseline.[Table tbl5]

**Figure 1 fig1:**
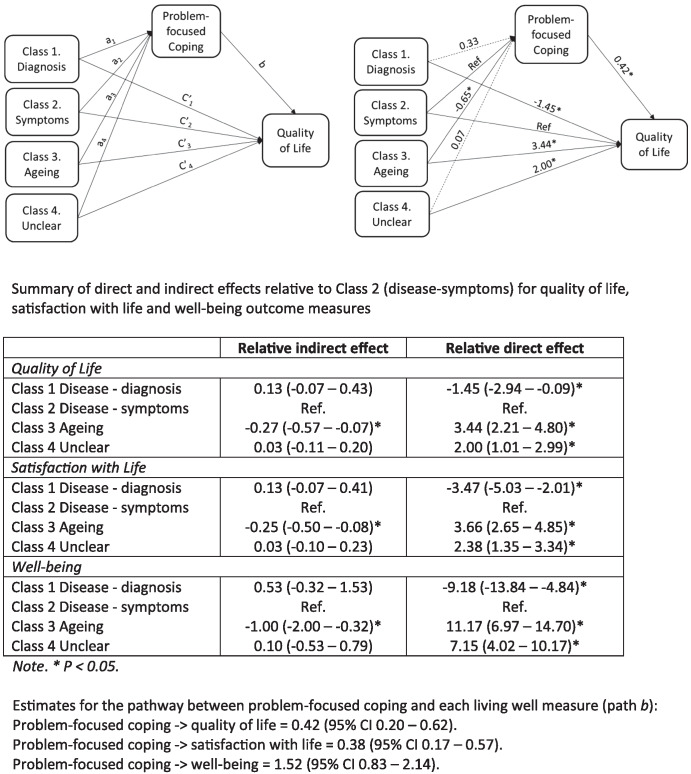
Problem-Focused Coping as a Mediator of the Relationship Between Class Membership and Quality of Life Score at Baseline *Note*. The model was replicated for satisfaction with life and well-being.

With regard to coping styles, the problem-focused coping score was selected for exploration of possible mediation effects because it both showed a statistically significant difference between the classes and, being based on responses to six items, was considered a sufficiently robust measure. To determine whether problem-focused coping acted as a mediator between group membership and “living well,” mediation analysis was conducted at baseline, using “disease-symptoms” as the reference group. As shown in Figure 1, problem-focused coping was positively associated with QoL (Path b). The coefficients for the direct paths between class membership and QoL (Path c) indicated higher QoL for the “aging” and “unclear” classes and lower QoL for the “disease-diagnosis class, relative to the ‘disease-symptoms” class. Coefficients for the indirect path for QoL via problem-focused coping (Path a × Path b) did not differ significantly from the “disease-symptoms” reference class for the “disease-diagnosis” and “unclear” classes but were lower for the “aging” class. This indicates that lower use of problem-focused coping was negatively impacting on QoL for the “aging” class. Greater use of problem-focused coping was associated with better QoL, but “aging” class members were less likely than others to use problem-focused coping. By attributing the symptoms to normal aging, the participants in the aging class were able to maintain better QoL despite the detrimental effects of reduced engagement in problem-focused actions aimed at minimizing the impact of their symptoms on QoL, though the negative impact on the overall level of QoL was small. Very similar patterns were seen for associations with satisfaction with life and well-being. Mediation analyses were also conducted using class at Time 1, problem-focused coping at Time 2, and living well measures at Time 3. Despite the reduced sample size resulting from incorporating multiple time points, results were similar to those at baseline, reflecting the limited change in “living well” over time (see Figure S2).[Fig fig1]

## Discussion

This is the first large-scale study to examine the DRs held by people diagnosed with mild-to-moderate dementia and the first to use a validated structured measure to explore DRs. Based on the theoretical framework of the CSM of illness regulation, DRs were elicited covering the core components of IRs and identified four distinct classes (“disease-diagnosis,” “disease-symptoms,” “aging,” and “unclear”), with significant differences across classes on all representation elements and perceived consequences; a further group (“no problem”) who did not acknowledge any difficulties was also identified. “Disease” representations were relatively more common among younger individuals. The first hypothesis, that those holding illness-type representations would score lower on measures of “living well” than other groups, was supported. The “disease-diagnosis” and “disease-symptoms” classes had higher cognitive test scores but lower scores for QoL, satisfaction with life, and well-being, which were generally stable over 24 months, as well as more negative attitudes to own aging, and higher scores for depressive symptoms, than the other groups. The second hypothesis, that there would be between-group differences in the coping styles adopted, was partially supported, with the most salient difference being lower endorsement of problem-focused coping strategies by participants in the “aging” class. For the “aging” class, problem-focused coping appeared to play a mediating role in the association between DR and scores on measures of “living well.”

The findings confirm that it is possible to elicit DRs reflecting perceptions of label, cause, control, timeline, and consequences and to identify groups sharing certain common patterns with regard to their DRs. Four groups were identified through latent class analysis, alongside the “no problem” group identified through screening. Our previous study with a smaller sample yielded three clusters, “illness,” “aging,” and “no problem” (Clare et al., 2016). All 12 studies included in a systematic review of studies using cluster analysis based on IRs in chronic health conditions yielded either two or three clusters (Rivera et al., 2020); however, sample sizes ranged from 44 to 227, with a mean of 115, so it is possible that larger sample sizes yield more fine-grained classifications. With our large sample, we were able to differentiate the equivalent of the “illness” cluster found in our earlier small study into two classes, containing those who did and did not use a diagnostic label to describe the disease, while the “aging” class and “no problem” group emerged as before. A novel development was the identification of the “unclear” class, characterized by uncertainty in particular about how the condition would develop over time.

For many participants, the DR diverged considerably from a medical or “expert” understanding of the nature of the condition. Some participants did have a clear representation, but one that reflected either expectations of aging or a lack of acknowledgment of difficulties. Participants in the “disease-diagnosis” group, who used a diagnostic label and mostly saw the condition as a brain disease which would get worse over time, constituted only 14% of the sample. In line with previous findings (Clare et al., 2016), relatively few participants from the other groups spontaneously offered a diagnostic label to describe the condition; even when prompted, the most frequent response as regards to cause was “don’t know,” and fewer than half expected the condition to get worse over time. It has been noted that lack of coherence in IRs is likely to have pervasive effects on coping and outcomes (Hagger & Orbell, 2021). DRs also diverged considerably from the beliefs held by caregivers. Among 1,277 caregivers in the IDEAL cohort, who included the 889 study partners of participants in the current analysis, over 90% of caregivers were aware of the diagnosis and able to state it when prompted, although only 49% spontaneously used this terminology, and 85% attributed the cause to changes in the brain or to a disease (Quinn et al., 2019).

IRs are conceptualized as memory structures accessed through cognitive processes in response to perceptions of a health threat. Dementia can be understood as a profound threat to identity and independence (Clare, 2003) that also prompts awareness of mortality. Perceptions of health threat give rise to cognitive and emotional processes that may serve at a psychological level to protect the self against the resulting anxiety or fear, and as dementia is a condition of later life that centrally involves changes in cognition, the possible impact of both age-related and dementia-related changes in cognitive and emotional processing on DRs should be considered. First, age-related changes in emotion regulation may help to account for some of our findings. Socioemotional selectivity theory posits that increasing age alters future time perspective, leading to changes in motivation, information processing, emotion regulation, and adoption of coping strategies (Carstensen et al., 2003), with selective attention to positive information and a greater focus on present experience. While these changes may benefit general well-being, they may be detrimental to managing health problems if people adapt their information seeking to avoid consideration of unpleasant possibilities in order to maintain positive mood in the present (Löckenhoff & Carstensen, 2004). This age-related shift in emotion regulation could help to explain why, although two-thirds of participants in the present study knew their diagnosis, fewer than half thought the condition would progress. Similarly, it might help to explain why as many as two-thirds of participants said there were things that they could do to control the effects of the condition or the difficulties they experienced. The qualitative data that contributed to developing the RADIX (Clare et al., 2016) indicated that alongside taking prescribed medication, perceptions of control included applying general strategies such as keeping one’s brain active (e.g., through doing puzzles) and using specific strategies to counter the effects of memory or other cognitive difficulties (e.g., writing things down, making lists). These kinds of strategies can potentially be used by any individual and may help maintain a positive emotional state.

Second, cognitive changes due to dementia may affect DRs. Because a decline in aspects of cognitive and functional ability is a defining feature of dementia (Martyr & Clare, 2012), DRs themselves or the processes through which they are activated may be affected by the intrinsic nature of dementia. It could be difficult for people to remember information they have been told or apply that knowledge to their own situation. This may be compounded by several related issues. Practice in communicating the diagnosis is variable (Lecouturier et al., 2008; Yates et al., 2021), and responses to communication of a diagnosis may be influenced by expectations about age-related change and attitudes to aging. Prevalent cultural attitudes reflected in disaster-related metaphors (“rising tide,” “tsunami,” “time-bomb”) that often outweigh more positive representations (Zeilig, 2014), and perceptions of stigma, may also affect responses (Burgener & Berger, 2008; World Health Organization, 2012).

Third, self-protective psychological processes may explain why, in some cases, the diagnosis of dementia or the associated difficulties are not acknowledged at a given time point (Clare et al., 2011). Lack of awareness, or anosognosia, is sometimes described as widespread among people with dementia (Lenzoni et al., 2020), but our study suggests otherwise (Clare et al., 2012; Martyr et al., 2011, 2012). Only 7% of participants responded to screening questions about dementia-related symptoms in a way that suggested either complete unwillingness to acknowledge any difficulties or a lack of awareness of any difficulties. Some individuals with mild-to-moderate dementia may be unable, due to neurological damage, to assimilate information about their condition and integrate this into an updated self-concept (Mograbi et al., 2009). However, most people with mild-to-moderate dementia have some awareness of the changes or difficulties they are experiencing but vary in how they construe these difficulties and in the accuracy with which they appraise aspects of their current functioning; these variations can be understood as resulting from the interaction of neurological, psychological, and social factors in each individual case (Clare et al., 2011, 2012). From a psychosocial perspective, the onset of dementia has been understood as a threat to selfhood, where dynamic processes such as avoidance, denial, or repression provide a means of self-preservation and coping (Clare, 2003; Sabat & Harré, 1992), and this may offer a particularly salient explanation as to why some people seem not to acknowledge the diagnosis or resulting difficulties in the mild-to-moderate stages of dementia. One psychological mechanism shown to underlie the processing and recall of self-threatening information in the general population is selective forgetting of self-threatening feedback, termed the mnemic neglect effect (Sedikides et al., 2016). There is evidence that people with dementia show this effect when asked to recall highly negative self-referent dementia-related information (Cheston et al., 2018). As this phenomenon is seen for recall but not for recognition memory, the information which is not directly recalled may have been processed implicitly, supporting other evidence that implicit awareness can affect behavior in the absence of any indication of explicit awareness (Martyr et al., 2011). These kinds of psychological processes may help to explain reluctance to use a diagnostic label even where this is known and can be elicited with direct prompting and may partly account for the observation that fewer than half the participants said they expected the condition to worsen over time. Hope of stability or improvement may be a means of maintaining psychological equilibrium.

If these responses are self-protective, it raises the question as to what constitutes a positive DR. Typically IR clusters are described as positive or negative based on their associations with health outcomes (Rivera et al., 2020). In the case of dementia, however, the issues are complex. People have a right to know their diagnosis, and a timely diagnosis is considered important in order to enable people to develop coping strategies and plan for the future. Yet participants in the disease clusters had lower scores than those in the “aging” and “no problem” groups for QoL, satisfaction with life, and well-being and higher scores for depressive symptoms. One possibility is that this could reflect a process of adjustment that occurs over time; reduced well-being might be linked to the initial impact of taking on board the diagnosis and its implications and might resolve given time. However, the present study suggest this is not the case, as the differences in outcomes between the groups remained the same 24 months later, and those in the “disease-diagnosis” group were not necessarily those most recently diagnosed. The importance of this issue is highlighted further by the fact that a similar pattern was seen among caregivers in the IDEAL cohort; those who attributed the cause to aging or said they did not know the cause had higher levels of well-being and lower stress levels than those who adopted a disease-based explanation (Quinn et al., 2019).

In terms of coping style, although differences were small, the most marked between-group difference was that people with dementia who held an “aging” model were less likely than other groups to use problem-focused coping. This may be because “aging” is a representation that tends to normalize any difficulties experienced and view them as a natural and expected part of growing older. While there is evidence that coping acts as a mediator of the associations between IRs and outcomes (Hagger et al., 2017), this appears to be only partially the case for DRs. Compared to the “disease-symptoms” class, for the “aging” and “unclear” classes, there was a direct association of class membership with scores on measures of “living well,” whereas the direct effects were lower for those in the “disease-diagnosis” class; indirect effects via problem-focused coping were similar for the “disease-diagnosis” and “unclear” classes, but lower for the “aging” class. This suggests that intervening to encourage use of problem-focused coping strategies may be beneficial in supporting positive outcomes, particularly for those with an “aging” representation, since they make less use of problem-focused coping and this has a negative impact on their QoL.

In this study, we tested a theoretical model with a large and well-characterized sample of people with mild-to-moderate dementia. Nevertheless, some limitations must be taken into account. Participants had all been given a medical diagnosis of dementia, but we do not know exactly how the diagnosis was communicated or what they were told about the condition or what information was communicated to family members. Practice in this regard is known to vary (Lecouturier et al., 2008; Yates et al., 2021), and this could affect the development of DRs. A high proportion of participants were unable to state their diagnosis, and while it is accepted good practice to communicate the diagnosis, we cannot be certain that everyone had been told. Equally we do not know exactly how prior knowledge about dementia may have shaped responses. In this article, we have not examined stability of DRs over time, but we plan to address this separately.

The focus of the present study was on the subjective experience of people with mild-to-moderate dementia, primarily using a range of self-report measures. Some items may have been challenging for participants with dementia, but the measures were administered in an interview context and this meant the researcher could take time over more challenging items and repeat them as often as needed to aid understanding. A previous study using IDEAL cohort data (Wu et al., 2020) comparing self- and informant ratings on the measures of “living well” used in the present study indicated that, while self-ratings by people with dementia were higher than informant ratings, the two sets of ratings had consistent relationships with a range of other factors. This demonstrates first that self-ratings are needed to understand subjective experience, and second that the self-ratings used in the present study can be considered equally as reliable as informant ratings when exploring associations with other factors.

Use of a validated structured measure of DRs is a strength. We did, however, observe missing data across all items, reaching levels >5% for control (9.2%), practical consequences (7.8%), and emotional consequences (6.8%). In this study, the RADIX was embedded in a relatively lengthy interview as part of a large survey, and items may have been missed for a variety of reasons. Given the nature of the RADIX and the type of reflection and processing required of participants, it may be preferable where possible to administer the measure in the context of a shorter, more focused interview. Measurement of coping followed the recommendation of Hagger and Orbell (2021) that coping should be considered in a disease-specific manner. As there is no specific measure of coping among people with dementia, items were developed from interviews with people with dementia that elicited qualitative accounts of coping, and suitability determined through expert rating and factor analysis. The resulting items reflected accepted definitions of key coping styles as well as being acceptable to participants; however, the items should not be seen as forming a validated scale. Coping styles were not evenly represented; half the items reflected aspects of problem-focused coping, with smaller numbers of items representing cognitive reappraisal, avoidance, and seeking social support. This meant there was limited variance in the three latter categories, and in particular the seeking social support category, which had only one item.

The evidence this study provides about the nature of DRs has a number of implications. First, the information people with dementia access about the condition and how professionals communicate diagnostic and other details require careful consideration. The way information is phrased and communicated may directly affect the development of DRs. The negative statements used to demonstrate the mnemic neglect effect among people with dementia (Cheston et al., 2018) were all taken from information leaflets widely available to people living with dementia in Great Britain, which are likely similar to those available in many other countries. Recall is better where statements describe another individual rather than the self, where information is delivered by a trusted familiar person, and where self-esteem is boosted first. This suggests that communication of the diagnosis and related details may be best done by a familiar and trusted health professional, that it is advisable to acknowledge a person’s strengths and capabilities before communicating difficult or potentially distressing information, and that explaining the condition and how to cope with it through the vehicle of personal stories or experiences narrated by individuals living with dementia might be particularly effective. Some people with dementia attest to the value of support gained through peer-to-peer networks where challenges are understood and experiences and coping strategies shared (Clare et al., 2008), so the benefits of peer-to-peer communication could be harnessed more widely.

Second, it has implications for the kinds of support and interventions that are offered to people living with dementia and their families. Identifying patterns in the ways in which people make sense of the condition offers one means of personalizing interventions to optimize their perceived relevance and benefits. For example, interventions aimed at encouraging problem-focused coping may help to improve QoL particularly for those with “aging” representations, while those with other kinds of representation who already engage in problem-focused coping may benefit more from different approaches to improving mood, self-efficacy, and perceived capability to “live well.” The same intervention may be received in different ways and could potentially be harmful for some while benefitting others. Therefore, with interventions such as self-management or support groups focused on coping with the condition, a single standard approach may not be optimal. Instead, the approach could be adjusted to meet the needs of groups or individuals with different DR profiles.

Third, it points to the importance of reflecting on what constitutes a positive or helpful DR. While the “disease-diagnosis” representation is most closely aligned with medical understanding, it also carries a detriment in terms of poorer QoL, satisfaction with life, and well-being. It would be inappropriate to actively encourage incorrect expectations, but the delicate balance between realism and maintaining hope requires careful navigation. Reaching consensus on the kinds of DRs likely to be most beneficial and hence most desirable to encourage among people with dementia and for their families, as well as on potential harms, would be helpful. Such a consensus would be important for shaping public education messages and campaigns aimed at raising awareness of dementia, and hence influencing prototype representations among those who might in time be diagnosed with dementia or find themselves caring for someone with dementia. Given the observation that currently available information can be perceived as highly negative (Cheston et al., 2018), finding an appropriate balance between acknowledging the reality of dementia and offering hope for living with dementia or supporting people with dementia would be crucial. Reflection on this issue could also add to wider debates about the impact of cultural metaphors of ill-health, such as the effect on patients with incurable conditions of using military (“fighting,” “war”) terminology (McCartney, 2014).

Finally, it is worthwhile considering whether, as the number of people living with dementia increases and policy, practice, and public awareness continue to evolve, the nature and distribution of DRs among people diagnosed with the condition might be shifting. There is some indication that certain elements of prototype representations held by the general public might be changing over time, with a reduction in the erroneous belief that there is a cure available and an increase in the belief that effective treatments exist seen over an 8 year period from 2008 to 2017, although no change in the extent to which dementia is viewed as part of the normal aging process (Alzheimer’s Disease International, 2019; Alzheimer’s Research UK, n.d.; Cations et al., 2018). Whether this corresponds to changes in how people with dementia view the condition is difficult to determine from available evidence. However, this could represent an opportunity to lay the groundwork for development of DRs associated with better capability to “live well” with the condition among those who may eventually be diagnosed with dementia through changes in public discourse resulting from effective public education campaigns. This could serve as a strategic goal for policy development.

## Conclusions

This study provides the first large-scale evidence that DRs can be reliably identified among people living with mild-to-moderate dementia and demonstrates the potential utility of understanding what type of DR a given individual holds. Distinct DR profiles can be discerned that are differentially associated with individual and disease-related characteristics and outcomes. Of note, representations diverging from medical understanding of the condition appear to be associated with better self-reported mood, QoL, satisfaction with life, and well-being. This raises questions about what constitutes a positive DR and how its development might be encouraged, and about the best way to support people with different DR profiles. Variations in DRs may reflect individual differences in the psychological processes involved in adjusting to the onset and progression of dementia, and these processes can be influenced by the ways in which information about the condition is communicated. DRs could offer a framework for personalizing and tailoring both the style and content of communications about dementia and intervention approaches aimed at supporting people in coping with dementia, in order to promote optimal benefits.

## Supplementary Material

10.1037/pag0000650.supp

## Figures and Tables

**Table 1 tbl1:** Characteristics of People With Dementia in the IDEAL Cohort Whose Data Were Included in the Analysis (N = 1,109)

Measure		Whole sample	
		*n*	%
Sex	Male	626	56.4
	Female	483	43.6
Age group	<65	103	9.3
	65–69	120	10.8
	70–74	188	17.0
	75–79	269	24.3
	80+	429	38.7
Age (*M*, *SD*)	76.4 (8.7)		
Ethnicity	White British	1,037	93.5
	Other	50	4.5
	Missing	22	2.0
Socioeconomic status	I (professional)	94	8.5
	II (managerial and technical)	380	34.3
	III-NM (skilled nonmanual)	212	19.1
	III-M (skilled manual)	223	20.1
	IV (partly skilled)	101	9.1
	V (unskilled)	23	2.1
	Not applicable	46	4.1
	Missing	16	1.4
	Armed forces	14	1.3
Education	No qualifications	303	27.3
	School leaving certificate at age 16	192	17.3
	School leaving certificate at age 18	352	31.7
	University	233	21.0
	Missing	29	2.6
Living situation	Living with spouse/partner	827	74.6
	Living with others	215	19.4
	Living alone	64	5.8
	Missing	3	0.3
Dementia type	AD	593	53.5
	VaD	125	11.3
	Mixed AD/VaD	244	22.0
	FTD	43	3.9
	PDD	32	2.9
	DLB	42	3.8
	Unspecified/other	30	2.7
When diagnosed	<1 year ago	595	53.7
	1–2 years ago	303	27.3
	3–5 years ago	100	9.0
	6+ years ago	16	1.4
	Missing	95	8.6
Years since diagnosis (*M*, *SD*)	0.95 (2.37)		
Caregiver in study	Spouse/partner	721	65.0
	Other family member/friend	168	15.1
	No caregiver participating in study	220	19.8
*Note*. IDEAL = Improving the experience of Dementia and Enhancing Active Life; AD = Alzheimer’s disease; VaD = vascular dementia; FTD = frontotemporal dementia; PDD = Parkinson’s disease dementia; DLB = dementia with Lewy bodies.			

**Table 2 tbl2:** Responses to the RADIX and Associated Coping Questions

(a) RADIX dementia representations for all participants with dementia who completed the RADIX (*n* = 1,033)					
Domain	*n*	%	Response	*n*	%
Identity (spontaneous description)	1,000	96.8	Diagnostic label	282	28.2
			Descriptive:		
			Symptoms	465	46.5
			Emotional	57	5.7
			General	21	2.1
			Aging	42	4.2
			No problem	21	2.1
			Don’t know	104	10.4
			Unclassifiable	8	0.8
Aware of specific diagnosis? (prompted if not stated spontaneously)	1,008	97.5	Yes	658^a^	65.3
			No	350	34.7
			Specific diagnosis stated	568^a^	56.3
Cause	1,030	99.6	Aging	230	22.3
			Changes in the brain	253	24.6
			Illness/disease	107	10.4
			Hereditary	67	6.5
			Lifestyle/life events	100	9.7
			Don’t know	262	25.4
			Unclassifiable	11	1.1
Timeline	982	95.0	Better	57	5.8
			Same	154	15.7
			Worse	443	45.1
			Unsure	328	33.4
Control	932	90.2	Strongly agree	63	6.8
			Agree	545	58.5
			Disagree	290	31.1
			Strongly disagree	34	3.6
			Total score	*M*	*SD*
Consequences—practical	953	92.2	Total 4 items (max 16)	9.6	2.1
Consequences—emotional	964	96.4	Total 5 items (max 20)	12.9	2.8

(b) Coping styles of participants with dementia who completed the RADIX (*n* = 1,033)					
Domain	*n*	%		*M*	*SD*
Problem focused	919	91.2	Total 6 items—range 12–24	17.9	2.1
Cognitive reappraisal	949	91.5	Total 2 items—range 2–8	6.2	0.8
Avoidance	952	92.2	Total 3 items—range 3–12	8.1	1.4
Seeking social support	981	95.0	Total 1 item—range 1–4	2.9	0.7
*Note*. RADIX = Representations and Adjustment to Dementia Index.					
^a^ Includes the 282 participants who spontaneously gave a diagnostic label in response to the identity question. When the 726 participants who did not spontaneously give a diagnostic label were asked about awareness of a diagnosis (or “what does the doctor call it?”), 376 (37.3% of the whole sample) said they were aware of a diagnosis, and 286 (28.4% of the whole sample) then stated a diagnosis.					

**Table 3 tbl3:** RADIX Response Profiles and Coping Scores for the Four Classes, With Statistical Comparisons

(a) RADIX response profiles for the four classes^a^					
Domain	Class 1 disease-diagnosis(*n* = 141.9, 13.7%)	Class 2 disease-symptoms(*n* = 504.4, 48.8%)	Class 3 aging(*n* = 117.2, 11.3%)	Class 4 unclear(*n* = 269.4, 26.1%)	Statisticalcomparison
Identity	^2,3,4^	^1,3^	^1,2,4^	^1,3^	χ^2^**(12) = 53.15,** ***p*** < **.001**
Diagnostic label	43.9 (4.2)	29.0 (3.8)	10.5 (2.6)	25.9 (3.7)	
Descriptive-symptoms	31.1 (3.9)	48.4 (4.2)	57.1 (4.2)	46.5 (4.2)	
Descriptive-emotional	11.7 (2.7)	5.6 (1.9)	0.0 (0.0)	5.1 (1.8)	
Don’t know	4.6 (1.8)	10.1 (2.5)	9.1 (2.4)	14.6 (3.0)	
Other	8.7 (2.4)	6.8 (2.1)	23.3 (3.5)	7.8 (2.3)	
Missing	3.2%	2.8%	4.4%	3.4%	
Cause	^2,3,4^	^1,3,4^	^1,2,4^	^1,2,3^	χ^2^**(9) = 494.67,** ***p*** < **.001**
Aging	4.4 (1.7)	21.3 (3.4)	100.0 (0.0)	0.0 (0.0)	
Brain/disease/hereditary	57.2 (4.2)	44.7 (4.2)	0.0 (0.0)	45.0 (4.2)	
Lifestyle/life events	12.9 (2.8)	9.2 (2.4)	0.0 (0.0)	13.1 (2.8)	
Don’t know/unclassifiable	25.4 (3.7)	24.8 (3.6)	0.0 (0.0)	41.8 (4.1)	
Missing	0.1%	0.4%	0.2%	0.2%	
Timeline	^2,3,4^	^1,3,4^	^1,2,4^	^1,2,3^	χ^2^**(9) = 787.50,** ***p*** < **.001**
Better	0.9 (0.8)	0.2 (0.4)	10.8 (2.6)	16.8 (3.1)	
Same	3.4 (1.5)	1.2 (0.9)	43.9 (4.2)	37.6 (4.1)	
Worse	75.8 (3.6)	68.2 (3.9)	02.6 (1.3)	3.3 (1.5)	
Unsure	19.9 (3.3)	30.4 (3.9)	42.7 (4.2)	42.3 (4.1)	
Missing	3.8%	4.6%	7.1%	5.3%	
Control	^2,3,4^	^1^	^1^	^1^	χ^2^**(9) = 66.45,** ***p*** < **.001**
Strongly agree	10.5 (2.6)	4.4 (1.7)	7.1 (2.2)	0.9 (0.8)	
Agree	41.2 (4.1)	61.1 (4.1)	63.0 (4.1)	61.3 (4.1)	
Disagree	33.5 (4.0)	32.7 (3.9)	29.9 (3.8)	27.3 (3.7)	
Strongly disagree	14.9 (3.0)	1.8 (1.1)	0.0 (0.0)	2.4 (1.3)	
Missing	5.4%	9.9%	13.7%	10.1%	
Diagnosis awareness	^2,3,4^	^1,3^	^1,2,4^	^1,3^	χ^2^**(3) = 62.57,** ***p*** < **0.001**
Yes aware	83.9 (3.1)	67.6 (3.9)	40.0 (4.1)	62.1 (4.1)	
No not aware	16.1 (3.1)	32.4 (3.9)	60.0 (4.1)	37.9 (4.1)	
Missing	1.5%	2.9%	1.6%	2.4%	
Diagnosis knowledge	^2,3,4^	^1,3^	^1,2,4^	^1,3^	χ^2^**(3) = 81.55,** ***p*** < **.001**
Diagnosis stated	81.5 (3.3)	59.3 (4.1)	31.3 (3.9)	50.2 (4.2)	
Diagnosis not stated	18.5 (3.3)	40.7 (4.1)	68.7 (3.9)	49.8 (4.2)	
Missing	1.7%	3.4%	2.5%	4.3%	
Consequences	Class 1 *M* (*SE*) range, % missing	Class 2 *M* (*SE*) range, % missing	Class 3 *M* (*SE*) range, % missing	Class 4 *M* (*SE*) range, % missing	Statistical comparison
Practical consequences	10.66 (0.25)^2,3,4^ 4–16, 5.6%	9.63 (0.10)^1,3^ 4–16, 7.9%	8.76 (0.16)^1,2^ 4–14, 10.3%	9.32 (0.14)^1^ 4–16, 7.4%	χ^2^**(3) = 44.89,** ***p*** < **.001**
Emotional consequences	14.12 (0.34)^2,3,4^ 5–20, 4.2%	13.00 (0.14)^1,3^ 5–20, 6.9%	11.42 (0.24)^1,2,4^ 5–19, 9.4%	12.51 (0.20)^1,3^ 5–20, 6.3%	χ^2^**(3) = 50.64,** ***p*** < **.001**
(b) Coping styles for the four classes^b^					
Coping strategies	Class 1 *M* (*SE*) range, % missing	Class 2 *M* (*SE*) range, % missing	Class 3 *M* (*SE*) range, % missing	Class 4 *M* (*SE*) range, % missing	Statistical comparison
Problem-focused	18.08 (0.25) 12–24, 9.2%	17.85 (0.11)^3^ 12–24, 10.5%	17.25 (0.21)^2,4^ 13–24, 12.0%	18.01 (0.15)^3^ 12–24, 12.6%	χ^2^**(3) = 10.62,** ***p*** = **.014**
Cognitive reappraisal	6.42 (0.10) 2–8, 7.1%	6.15 (0.04) 4–8, 7.9%	6.13 (0.07) 5–8, 10.1%	6.11 (0.06) 2–8, 8.3%	χ^2^(3) = 7.59, *p* = .055
Avoidant	8.10 (0.17) 3–12, 6.7%	8.22 (0.07) 3–12, 7.2%	8.17 (0.14) 6–12, 11.3%	7.96 (0.10) 3–12, 8.2%	χ^2^(3) = 3.77, *p* = .287
Seeking social support	2.95 (0.08) 1–4, 4.5%	2.91 (0.03) 1–4, 5.0%	2.73 (0.07) 1–4, 5.4%	2.75 (0.05) 1–4, 5.2%	χ^2^**(3) = 11.70,** ***p*** = **.008**
*Note*. RADIX = Representations and Adjustment to Dementia Index.					
^a^ Categorical variables are presented as percentages (*SE*), and continuous variables as *M* (*SE*). Bold chi-square test results indicate significance at the 5% level after Holm–Bonferroni correction. Where significant, post hoc comparisons between each class were reported if *p* < .004, and these significant differences are denoted by numbered superscripts which correspond with class number. For example, under the results for Class 1, superscripts 2, 3, and 4 indicate that the mean for the given variable was significantly different from Classes 2, 3, and 4 after Bonferroni correction. ^b^ Bold chi-square test results indicate significance at the 5% level after Holm–Bonferroni correction. Where significant, post hoc comparisons between each class were reported if *p* < .008, and these significant differences are denoted by numbered superscripts which correspond with class number. For example, under the results for Class 1, superscripts 2, 3, and 4 would indicate that the mean for the given variable was significantly different from Classes 2, 3, and 4 after Bonferroni correction.					

**Table 4 tbl4:** Multinomial Regression Examining Predictors of Group Membership Across the Four Classes and the “No Problem” Group, With Class 2 (Disease-Symptoms) as the Reference Category

Dementia representation	Model 1. Univariable	Model 2. Multivariable model
Age		
Class 1. Disease-diagnosis	0.959 (0.941–0.978)*	0.941 (0.916–0.967)*
Class 3. Aging	1.107 (1.079–1.135)*	1.128 (1.083–1.175)*
Class 4. Unclear	1.007 (0.991–1.022)	1.007 (0.986–1.030)
No problem	1.040 (1.004–1.078)*	1.043 (0.985–1.105)
Dementia subtype (AD/VaD/Mixed AD/VaD vs. Other)		
Class 1. Disease-diagnosis	0.691 (0.441–1.083)	0.701 (0.401–1.227)
Class 3. Aging	1.730 (0.891–3.361)	0.819 (0.358–1.870)
Class 4. Unclear	1.065 (0.723–1.567)	0.881 (0.539–1.438)
No problem	1.018 (0.502–2.066)	0.536 (0.187–1.534)
Time since diagnosis (years)		
Class 1. Disease-diagnosis	1.028 (0.956–1.104)	1.019 (0.961–1.080)
Class 3. Aging	0.887 (0.745–1.056)	0.811 (0.644–1.020)
Class 4. Unclear	0.962 (0.893–1.035)	0.939 (0.860–1.025)
No problem	1.019 (0.947–1.097)	1.005 (0.898–1.125)
Cognitive ability (ACE-III)		
Class 1. Disease-diagnosis	1.013 (0.998–1.029)	1.002 (0.985–1.019)
Class 3. Aging	0.982 (0.969–0.995)*	0.976 (0.957–0.996)*
Class 4. Unclear	0.985 (0.975–0.995)*	0.976 (0.963–0.998)*
No problem	0.971 (0.954–0.987)*	0.962 (0.940–0.985)*
Functional ability (FAQ-I)		
Class 1. Disease-diagnosis	0.986 (0.967–1.005)	—
Class 3. Aging	1.004 (0.980–1.029)	—
Class 4. Unclear	1.006 (0.988–1.024)	—
No problem	1.036 (0.997–1.076)	—
Comorbidity (CCI)		
Class 1. Disease-diagnosis	1.073 (0.981–1.174)	1.207 (1.083–1.345)*
Class 3. Aging	1.108 (1.034–1.186)*	0.984 (0.845–1.146)
Class 4. Unclear	1.025 (0.963–1.091)	1.080 (0.988–1.182)
No problem	1.031 (0.916–1.161)	1.119 (0.915–1.368)
Attitudes Toward Own Aging		
Class 1. Disease-diagnosis	0.878 (0.785–0.983)*	1.031 (0.875–1.214)
Class 3. Aging	1.268 (1.128–1.398)*	1.190 (0.993–1.425)
Class 4. Unclear	1.205 (1.109–1.309)*	1.129 (0.992–1.285)
No problem	1.788 (1.509–2.118)*	1.288 (0.959–1.731)
Self-efficacy		
Class 1. Disease-diagnosis	0.957 (0.925–0.990)*	0.986 (0.945–1.028)
Class 3. Aging	1.059 (1.023–1.091)*	1.026 (0.974–1.081)
Class 4. Unclear	1.043 (1.019–1.065)*	1.050 (1.016–1.084)*
No problem	1.108 (1.046–1.163)*	1.079 (0.994–1.172)
Depression (GDS-10)		
Class 1. Disease-diagnosis	1.127 (1.053–1.206)*	1.046 (0.944–1.159)
Class 3. Aging	0.849 (0.779–0.926)*	0.877 (0.763–1.008)
Class 4. Unclear	0.907 (0.854–0.963)*	0.947 (0.863–1.039)
No problem	0.632 (0.517–0.772)*	0.701 (0.486–1.012)
*Note*. AD = Alzheimer’s disease; VaD = vascular dementia; ACE-III = Addenbrooke’s Cognitive Examination-III; CCI = Charlson Comorbidity Index; FAQ-I = Functional Activities Questionnaire-Informant rating; GDS-10 = Geriatric Depression Scale 10-item version. The “no problem” group comprised 76 people who responded negatively to all Representations and Adjustment to Dementia Index (RADIX) screening questions, indicating that they perceived no difficulties. For the multivariable model, 727 participants had available data on all covariates. The multivariable model combines all the variables from Model 1, except for informant-rated functional ability; for this measure, data were only available for those individuals with a participating caregiver.		
* *p* < 0.05.		

**Table 5 tbl5:** Association Between Group Membership and “Living Well” Scores Across Time Points, With Class 2 (Disease-Symptoms) as the Reference Group

Outputs from latent growth curve models	QoL-AD unadjusted	QoL-AD adjusted^a^	SwLS unadjusted	SwLS adjusted^a^	WHO-5 unadjusted	WHO-5 adjusted^a^
Intercept	35.67 (35.12–36.23)*	33.92 (30.61–37.23)*	25.14 (24.58–25.69)*	22.34 (19.53–26.26)*	57.85 (55.98–59.73)*	51.81 (40.46–63.16)*
Slope	0.01 (−0.31 to 0.32)	0.15 (−1.81 to 2.11)	0.24 (−0.10 to 0.58)	−1.15 (−3.28 to 1.02)	0.35 (−0.81 to 1.52)	5.21 (−2.18 to 12.59)
Intercept on Class 1 diagnosis	−1.69 (−2.93 to −0.44)*	−1.47 (−2.69 to −0.25)*	−3.53 (−4.76 to −2.30)*	−3.30 (−4.53 to −2.08)*	−8.88 (−13.08 to −4.68)*	−8.24 (−12.40 to −4.07)*
Intercept on Class 2 symptoms	Ref	Ref	Ref	Ref	Ref	Ref
Intercept on Class 3 aging	2.99 (1.69–4.28)*	2.50 (1.20–3.80)*	3.34 (2.05–4.63)*	2.73 (1.43–4.03)*	10.04 (5.69–14.39)*	8.09 (3.72–12.47)*
Intercept on Class 4 unclear	2.19 (1.20–3.17)*	1.90 (0.94–2.87)*	2.39 (1.40–3.38)*	2.19 (1.21–3.17)*	7.12 (3.77–10.48)*	6.25 (2.94–9.55)*
Intercept on Group 5 no problem	5.29 (3.85–6.74)*	4.77 (3.35–6.19)*	3.90 (2.47–5.33)*	3.39 (1.98–4.81)*	13.38 (8.56–18.19)*	11.44 (6.68–16.21)*
Slope on Class 1 diagnosis	0.12 (−0.57 to 0.80)	0.11 (−0.58 to 0.79)	0.15 (−0.57 to 0.88)	0.32 (−0.42 to 1.05)	0.67 (−1.83 to 3.16)	0.43 (−2.11 to 2.98)
Slope on Class 2 symptoms	Ref	Ref	Ref	Ref	Ref	Ref
Slope on Class 3 aging	−0.38 (−1.10 to 0.35)	−0.47 (−1.23 to 0.28)	−0.31 (−1.11 to 0.48)	−0.45 (−1.27 to 0.36)	−2.65 (−5.36 to 0.07)	−2.39 (−5.20 to 0.43)
Slope on Class 4 unclear	−0.16 (−0.74 to 0.41)	−0.21 (−0.79 to 0.37)	−0.69 (−1.31 to −0.07)*	−0.66 (−1.28 to −0.04)*	−1.80 (−3.92 to 0.32)	−1.80 (−3.94 to 0.34)
Slope on Group 5 no problem	−0.66 (−1.54 to 0.22)	−0.73 (−1.61 to 0.16)	−0.63 (−1.61 to 0.35)	−0.61 (−1.59 to 0.37)	−2.73 (−6.05 to 0.60)	−2.58 (−5.92 to 0.76)
Residual intercept variance	25.32 (21.31–29.34)*	23.13 (19.31–26.96)*	23.49 (19.17–27.82)	22.06* (18.37–26.26)	282.62 (233.95–331.28)*	262.24 (215.15–309.34)*
Residual slope variance	2.56 (0.75–4.36)*	2.23 (0.47–3.98)*	1.84 (−0.40 to 4.08)	1.52 (−0.64 to 3.73)	38.54 (14.34–62.74)*	35.53 (11.57–59.50)*
*Note*. QoL-AD = Quality of Life in Alzheimer’s Disease; SwLS = Satisfaction with Life Scale, WHO-5 = World Health Organization-Five Well-Being Index.						
^a^ Adjusted for age, sex, and dementia subtype; The “no problem” group consisted of 76 people who responded negatively to all Representations and Adjustment to Dementia Index (RADIX) screening questions, indicating that they perceived no difficulties. Full information maximum likelihood (FIML) estimation was used to account for missing data. The model diagram for this analysis is shown in Figure S1. For estimation of the slope at least two time points are required. There were 748 participants with at least two quality of life measurements, 748 with at least two satisfaction with life measurements, and 815 with at least two well-being measurements. As shown in Table S7, the class compositions remain similar for those participants with two or more time points compared with the total sample.						
* *p* < 0.05.
