# Supplementary material for: Psychological Processes in Adapting to Dementia: Illness Representations Among the IDEAL Cohort
Source: Psychol Aging. 2021 Dec 9;37(4):524–41. doi: 10.1037/pag0000650 (PMC9134708; doi:10.1037/pag0000650)
Supplement: Supplementary file 1 [file PAG-2021-1227_Suppl.docx]

**Psychological processes in adapting to dementia: illness representations among the IDEAL cohort**

**SUPPLEMENTAL ONLINE FILE**

Linda Clare*^1,2^, Laura D. Gamble^3^, Anthony Martyr^1^, Catherine Quinn^4,5^, Rachael Litherland^6^, Robin G. Morris^7^, Ian R. Jones^8^, and Fiona E. Matthews^3^

1. REACH, College of Medicine and Health, University of Exeter, UK
2. NIHR Applied Research Collaboration South-West Peninsula, UK
3. Population Health Sciences Institute, Newcastle University. UK
4. Centre for Applied Dementia Studies, Bradford University, UK
5. Wolfson Centre for Applied Health Research, Bradford, UK
6. Innovations in Dementia CIC, Exeter, UK
7. Institute of Psychiatry, Psychology and Neuroscience, King’s College London, UK
8. Wales Institute for Social and Economic Research and Data, Cardiff University, UK

*Corresponding author:

Professor Linda Clare, Centre for Research in Ageing and Cognitive Health (REACH), College of Medicine and Health, St Luke’s Campus, University of Exeter, Exeter EX1 2LU, United Kingdom. Email: [l.clare@exeter.ac.uk](mailto:l.clare@exeter.ac.uk)

**ORCIDs:**

Linda Clare: 0000-0003-3989-5318

Laura D. Gamble: 0000-0001-8496-9705

Anthony Martyr: 0000-0002-1702-8902

Catherine Quinn: 0000-0001-9553-853X

Robin G. Morris: 0000-0001-7767-5258

Ian Rees Jones: 0000-0002-1682-9134

Fiona E. Matthews: 0000-0002-1728-2388

**Table S1**

*Reasons for attrition across the 3 waves*

| Status | Time 1 | Time 2 | Time 3 |
| --- | --- | --- | --- |
| In study | 1537 | 1183 | 851 |
| In study but did not take part at this time point | 8 | 12 | - |
| Died | - | 48 | 72 |
| Withdrew: health reasons | - | 98 | 117 |
| Withdrew: carer ill-health/carer not available | - | 13 | 6 |
| Withdrew: felt too burdensome/experienced bereavement | - | 58 | 55 |
| Withdrew: no longer interested | - | 47 | 25 |
| Withdrew: moved to care home or out of the area | - | 7 | 6 |
| Passive withdrawal: lost to follow up | - | 79 | 63 |
| Withdrew at previous time point | - | - | 350 |

**Table S2**

*Items used to assess coping style with details of the classification by expert raters and results of exploratory factor analysis*

| RADIX item | Category+ | Factor 1 | Factor 2 | Factor 3 | Factor 4 |
| --- | --- | --- | --- | --- | --- |
| I try to find practical ways of overcoming problems resulting from my x* | PF | **0.626** |  |  |  |
| There’s a lot I can do about my x; I’m going to fight it as long as I can | PF | **0.597** |  | -0.236 | -0.128 |
| I am keen to learn more about my x | PF | **0.538** |  | 0.127 | -0.317 |
| It helps to keep myself busy | PF | **0.492** | -0.113 | -0.289 | -0.289 |
| I find it helps to keep t a routine | PF | **0.461** | -0.22 |  |  |
| I give myself time and try to be patient with myself | PF | **0.388** |  | -0.124 | 0.137 |
| Due to my x I have to accept the changes in my life | CR | **0.521** | -0.248 | 0.326 | 0.208 |
| My x is not that bad; there are others worse off than me | CR | **0.367** | 0.241 |  | 0.301 |
| I try to hide the difficulties resulting from my x | AV |  | **0.72** | 0.121 |  |
| I try to avoid social contact because of my x | AV | 0.361 | **0.538** |  | 0.21 |
| I prefer not to talk about my x | AV | 0.19 | **0.326** | 0.305 | -0.189 |
| I rely on others for help | SS |  | -0.296 | **0.512** | 0.145 |
| I avoid thinking about my x^ | AV |  | 0.221 | 0.294 | **-0.348** |

*Note.* RADIX Representations and Adjustment to Dementia Index. +Category into which the item was classified by 5 expert raters. *In line with the approach taken in the RADIX, the interviewer employed the term used by the participant to describe the condition or the associated difficulties (e.g. Alzheimer’s, dementia, memory problems). ^Item removed from further analysis based on results of factor analysis

**Table S3**

*Model fit indices for 2-5 class latent class analysis solutions using identity, cause, timeline and control*

|  | **2-class** | **3-class** | **4-class** | **5-class** |
| --- | --- | --- | --- | --- |
| Parameters | 39 | 59 | 79 | 99 |
| Log-likelihood | -6236 | -6069 | -6043 | -6018 |
| BIC | 12743 | 12548 | 12635 | 12721 |
| ssBIC | 12620 | 12361 | 12383 | 12406 |
| Entropy | 0.829 | 0.777 | 0.819 | 0.734 |
| Class 1  Class 2  Class 3  Class 4  Class 5 | 17.0%  83.0% | 14.7%  49.6%  36.2% | 26.1%  13.7%  48.8%  11.3% | 12.8%  6.2%  46.8%  17.1%  17.1% |
| LMR-LRT | P < 0.001 | P < 0.001 | P = 0.008 | P = 0.664 |
| BLRT | P < 0.001 | P < 0.001 | P < 0.001 | P = 0.020 |

*Note.* BIC – Bayesian Information Criterion; ssBIC – sample size adjusted BIC, LMR-LRT – Lo-Mendell-Rubin likelihood ratio test, BLRT – bootstrapped likelihood ratio test.

**Table S4**

*Distributions of the 4 latent classes for categorical identity and cause variables, and for ordinal timeline and control variables*

|  | | **Class 1.**  **Disease - diagnosis**  **(13.7%,**  **N = 141.9)** | **Class 2.**  **Disease - symptoms**  **(48.8%,**  **N = 504.4)** | **Class 3.**  **Ageing**  **(11.3%,**  **N = 117.2)** | **Class 4.**  **Unclear**  **(26.1%,**  **N = 269.4)** |
| --- | --- | --- | --- | --- | --- |
| Identity | Diagnostic label  Descriptive – Symptoms  Descriptive - Emotional  Don’t know  Other | 0.439  0.311  0.117  0.046  0.087 | 0.290  0.484  0.056  0.100  0.068 | 0.105  0.571  0.000  0.091  0.233 | 0.259  0.465  0.051  0.146  0.078 |
| Cause | Ageing  Brain/Illness/hereditary  Lifestyle/life events  Don’t know/ unclassifiable | 0.044  0.572  0.129  0.254 | 0.213  0.447  0.092  0.248 | 1.000  0.000  0.000  0.000 | 0.000  0.450  0.131  0.418 |
| Timeline 1. My [label] will stay the same as it is now | Strongly agree  Agree  Disagree  Strongly disagree | 0.063  0.098  0.262  0.577 | 0.000  0.280  0.698  0.022 | 0.108  0.696  0.196  0.000 | 0.047  0.637  0.284  0.032 |
| Timeline 2. Me [label] will get better | Strongly agree  Agree  Disagree  Strongly disagree | 0.021  0.015  0.084  0.879 | 0.000  0.034  0.887  0.079 | 0.019  0.381  0.541  0.058 | 0.044  0.397  0.540  0.018 |
| Timeline 3. My [label] will get worse | Strongly disagree  Disagree  Agree  Strongly agree | 0.079  0.018  0.280  0.624 | 0.000  0.000  0.960  0.050 | 0.065  0.778  0.157  0.000 | 0.056  0.769  0.144  0.031 |
| Control. There is a lot I can do to control my [label] | Strongly disagree  Disagree  Agree  Strongly agree | 0.149  0.335  0.412  0.105 | 0.018  0.327  0.611  0.044 | 0.000  0.299  0.630  0.071 | 0.024  0.273  0.613  0.090 |

**Table S5**

*Characteristics and scores on study measures for the participants in the four classes and the ‘no problem’ group, and statistical comparisons*

1. *Demographic characteristics*

| Measure | Class 1  Disease –diagnosis  (13.7%) | Class 2  Disease – symptoms  (48.8%) | Class 3  Ageing  (11.3%) | Class 4  Unclear  (26.1%) | No problem (NP)  (6.9%) | Statistical comparison |
| --- | --- | --- | --- | --- | --- | --- |
| Sex: n=1109 (100.0%)  Male (56.4%)  Female (43.6%) | 54.6 (5.1)  45.4 (5.1) | 54.6 (2.5)  45.4 (2.5) | 49.7 (4.9)  50.3 (4.9) | 62.5 (3.3)  37.5 (3.3) | 60.5 (5.6)  39.5 (5.6) | $\chi^{2}$ (4) = 6.36, P = 0.174 |
| Age group: n=1109 (100.0%)  <65 (9.3%)  65-69 (10.8%)  70-74 (17.0%)  75-79 (24.3%)  80+ (38.7%)  Age (mean, SE) | ^3,4,NP^  19.7 (3.7)  20.5 (3.9)  20.0 (4.0)  16.8 (3.5)  23.0 (3.9)  72.4 (9.7) ^2,3,4,NP^ | ^3,NP^  9.6 (1.6)  11.4 (1.7)  21.7 (2.2)  27.0 (2.4)  30.2 (2.7)  76.0 (8.2) ^1,3^ | ^1,2,4,NP^  0.0 (0.0)  3.5 (1.5)  4.3 (1.9)  23.4 (3.7)  68.8 (4.1)  81.7 (6.3) ^1,2,4,NP^ | ^1,3^  9.1 (2.3)  9.1 (2.1)  16.1 (2.8)  28.1 (3.4)  37.7 (3.7)  76.3 (8.4) ^1,3^ | ^1,2,3^  7.9 (3.1)  10.5 (3.5)  13.2 (3.9)  11.8 (3.7)  56.6 (5.7)  78.5 (9.2) ^1,3^ | $\boldsymbol{\chi}^{\boldsymbol{2}}$ **(16) = 256.98, P < 0.001**  $\boldsymbol{\chi}^{\boldsymbol{2}}$ **(4) = 100.89, P < 0.001** |
| Socio-economic status: n = 1109 (100.0%)  I/II (professional/managerial and technical) (42.8%)  III-NM/III-M (skilled non-manual/manual) (39.2%)  IV (partly skilled/unskilled/armed forces) (12.5%)  Not applicable/Missing (5.5%) | 46.5 (4.7)  36.6 (4.7)  10.9 (3.3)  6.1 (2.3) | 44.8 (2.5)  38.3 (2.5)  12.4 (1.6)  4.5 (1.0) | 45.0 (4.9)  34.5 (4.7)  12.0 (3.2)  8.5 (2.8) | 38.1 (3.3)  42.2 (3.6)  12.9 (2.3)  6.9 (1.7) | 35.5 (5.5)  47.4 (5.7)  14.5 (4.0)  2.6 (1.8) | $\chi^{2}$ (32) = 10.59, P = 0.564 |
| Education: n=1080 (97.4%)  No qualifications (28.1%)  School leaving certificate at age 16 (17.8%)  School leaving certificate at age 18 (32.6%)  University (21.6%)  Missing | 26.7 (4.3)  18.1 (3.7)  29.2 (4.7)  26.1 (4.5)  1.9% | 26.1 (2.2)  16.9 (1.9)  32.5 (2.4)  24.5 (2.2)  2.1% | 37.1 (4.8)  18.7 (4.0)  27.8 (4.5)  16.4 (3.7)  2.2% | 27.8 (3.1)  18.6 (2.7)  36.6 (3.3)  17.0 (2.6)  2.6% | 30.0 (5.5)  18.6 (4.6)  32.9 (5.6)  18.6 (4.6)  7.9% | $\chi^{2}$ (12) = 11.46, P = 0.490 |
| Dementia type: n=1109 (100.0%)  AD (53.5%)  VaD (11.3%)  Mixed AD/VaD (22.0%)  FTD (3.9%)  PDD (2.9%)  DLB (3.8%)  Unspecified/other (2.7%) | ^3,4^  44.8 (4.7)  16.6 (3.4)  14.2 (3.4)  6.9 (2.3)  2.2 (1.3)  4.1 (1.7)  11.3 (2.9) | ^3,4^  52.6 (2.5)  11.5 (1.6)  20.9 (2.0)  2.2 (0.8)  4.5 (1.0)  4.0 (1.0)  4.2 (1.1) | ^1,2^  57.2 (4.9)  8.6 (2.8)  25.9 (4.4)  1.7 (1.2)  0.0 (0.0)  2.4 (1.5)  4.2 (2.0) | ^1,2^  57.0 (3.4)  11.5 (2.2)  19.5 (2.8)  5.2 (1.5)  2.0 (1.0)  4.9 (1.4)  0.0 (0.0) | 60.5 (5.6)  6.6 (2.8)  17.1 (4.3)  9.2 (3.3)  1.3 (1.3)  1.3 (1.3)  3.9 (2.2) | $\boldsymbol{\chi}^{\boldsymbol{2}}$ **(24) = 113.36, P < 0.001** |
| Diagnosed: n=1014 (91.4%)  < 1 year ago (58.7%)  1 – 2 years ago (29.9%)  3 – 5 years ago (9.9%)  6+ years ago (1.6%)  Years since diagnosis (mean, SE)  Missing | 52.2 (5.3)  29.0 (4.5)  12.3 (3.3)  6.4 (2.3)  1.31 (0.2.4)  8.1% | 57.9 (2.5)  32.5 (2.3)  8.8 (1.4)  0.8 (0.5)  0.97 (0.15)  7.7% | 68.9 (4.8)  23.9 (4.4)  5.5 (2.4)  1.7 (1.3)  0.66 (0.16)  8.4% | 61.3 (3.6)  26.5 (3.1)  11.2 (2.3)  1.0 (0.7)  0.81 (0.10)  8.5% | 50.0 (6.2)  34.4 (5.9)  15.6 (4.5)  0.0 (0.0)  1.11 (0.19)  15.8% | $\boldsymbol{\chi}^{\boldsymbol{2}}$ **(12) = 29.80, P = 0.003**  $\chi^{2}$ (4) = 7.43, P = 0.115 |
| Carer status: n=1109 (100%)  Spouse/partner  Family/friend  No carer involved | 69.8 (4.4)  13.2 (3.2)  17.0 (3.6) | 64.8 (2.4)  14.0 (1.7)  21.2 (2.0) | 53.2 (4.8)  27.8 (4.4)  19.0 (3.9) | 70.2 (3.5)  11.0 (2.5)  18.8 (2.9) | 63.2 (5.5)  15.8 (4.2)  21.1 (4.7) | $\chi^{2}$ (8) = 12.66, P = 0.124 |
| Living situation: n=1106 (99.7%)  Living alone  Living with spouse/partner  Lives with other  Missing | 19.0 (3.7)  75.6 (4.1)  5.4 (2.0)  0.4% | 19.8 (2.0)  75.9 (2.1)  4.3 (1.0)  0.2% | 24.2 (4.2)  66.2 (4.7)  9.6 (2.9)  0.0% | 16.3 (2.6)  77.2 (2.9)  6.5 (1.6)  0.4% | 21.1 (4.7)  71.1 (5.2)  07.9 (3.1)  0.0% | $\chi^{2}$ (8) = 7.66, P = 0.467 |

*Note.* AD Alzheimer’s disease; VaD vascular dementia; FTD frontotemporal dementia; PDD Parkinson’s disease dementia; DLB dementia with Lewy bodies.

Categorical outcome variables are presented as percentages (SE), and continuous variables as mean (SE). Bold indicates significance at the 5% level after Holm-Bonferroni correction. Where significant, post hoc comparisons between each class were reported if P < 0.0025, and the significant differences are denoted by numbered superscripts which correspond with class number or no problem group (NP). For example, under the results for Class 1, superscripts 2, 3 and 4 indicate that the mean for the given variable was significantly different from Classes 2, 3 and 4 after Bonferroni correction.

1. *Scores on study variables for participants with dementia*

| Measure | Class 1 | Class 2 | Class 3 | Class 4 | No problem (NP) | Comparison |
| --- | --- | --- | --- | --- | --- | --- |
|  | Mean (SE)  % missing | Mean (SE)  % missing | Mean (SE)  % missing | Mean (SE)  % missing | Mean (SE)  % missing |  |
| ACE-III total | 72.63 (1.42) ^3,4,NP^  7.8% | 70.43 (0.67) ^NP^  7.3% | 66.13 (1.42) ^1^  8.5% | 67.07 (0.95) ^1^  20.5% | 64.66 (1.61) ^1,2^  14.5% | $\boldsymbol{\chi}^{\boldsymbol{2}}$ **(4) = 25.48, P < 0.001** |
| CCI | 7.23 (0.30)  6.6% | 6.74 (0.11)  7.9% | 7.36 (0.20)  9.6% | 6.91 (0.16)  6.1% | 6.93 (0.28)  10.5% | $\chi^{2}$ (4) = 8.13, P = 0.087 |
| FAQ-I | 16.41 (0.83)  21.8% | 17.35 (0.51)  27.2% | 17.83 (1.01)  24.8% | 18.13 (0.71)  27.7% | 20.00 (1.24)  27.6% | $\chi^{2}$ (4) = 6.57, P = 0.160 |
| NPI-Q symptoms | 3.94 (0.24)  20.8% | 3.53 (0.14)  24.5% | 3.57 (0.26)  24.3% | 3.56 (0.21)  25.4% | 3.30 (0.30)  26.3% | $\chi^{2}$ (4) = 3.18, P = 0.529 |
| NPI-Q severity | 7.14 (0.55)  31.7% | 6.51 (0.30)  35.1% | 5.96 (0.54)  34.2% | 6.43 (0.43)  38.5% | 6.17 (0.56)  38.2% | $\chi^{2}$ (4) = 2.85, P = 0.583 |
| ATOA | 1.78 (0.15) ^2,3,4,NP^  5.6% | 2.21 (0.08) ^1,3,4,NP^  3.0% | 2.83 (0.16) ^1,2,NP^  3.4% | 2.77 (0.11) ^1,2,NP^  1.5% | 3.58 (0.16) ^1,2,3,4^  1.3% | $\boldsymbol{\chi}^{\boldsymbol{2}}$ **(4) = 93.07, P < 0.001** |
| Self-efficacy | 26.77 (0.71) ^3,4,NP^  7.0% | 28.72 (0.28) ^3,4,NP^  8.5% | 30.48 (0.50) ^1,2^  8.5% | 30.24 (0.34) ^1,2^  5.9% | 31.47 (0.65) ^1,2^  10.5% | $\boldsymbol{\chi}^{\boldsymbol{2}}$ **(4) = 39.74, P < 0.001** |
| Stigma | 7.68 (0.22)  8.3% | 7.71 (0.09)  8.6% | 7.14 (0.20)  9.0% | 7.64 (0.12)  8.0% | - | $\chi^{2}$ (3) = 6.70, P = 0.082 |
| GDS-10 | 3.84 (0.26) ^2,3,4,NP^  4.2% | 2.90 (0.13) ^1,3,NP^  3.2% | 2.12 (0.20) ^1,2,NP^  12.5% | 2.33 (0.16) ^1,NP^  8.3% | 1.28 (0.20) ^1,2,3,4^  11.8% | $\boldsymbol{\chi}^{\boldsymbol{2}}$ **(4) = 80.71, P < 0.001** |
| QoL-AD | 34.31 (0.70) ^3,4,NP^  12.0% | 35.75 (0.30) ^3,4,NP^  9.4% | 38.72 (0.58) ^1,2^  12.0% | 37.68 (0.38) ^1,2,NP^  7.8% | 40.96 (0.63) ^1,2,4^  10.5% | $\boldsymbol{\chi}^{\boldsymbol{2}}$ **(4) = 83.16, P < 0.001** |
| SwLS | 21.99 (0.72) ^2,3,4,NP^  1.4% | 25.16 (0.30) ^1,3,4,NP^  2.1% | 28.29 (0.53) ^1,2^  2.6% | 27.42 (0.39) ^1,2^  3.0% | 28.97 (0.57) ^1,2^  0.1% | $\boldsymbol{\chi}^{\boldsymbol{2}}$ **(4) = 94.24, P < 0.001** |
| WHO-5 | 49.32 (2.22) ^2,3,4,NP^  3.5% | 58.26 (1.04) ^1,3,4,NP^  1.8% | 67.33 (1.74) ^1,2^  0.0% | 65.16 (1.26) ^1,2^  1.5% | 71.16 (2.25) ^1,2^  0.0% | $\boldsymbol{\chi}^{\boldsymbol{2}}$ **(4) = 76.72, P < 0.001** |

*Note.* ACE-III Addenbrooke’s Cognitive Examination-III; ATOA Attitudes towards own ageing; CCI Charlson Comorbidity Index; FAQ-I Functional Activities Questionnaire-Informant rating; GDS-10 Geriatric Depression Scale 10-items; NPI-Q Neuropsychiatric Inventory Questionnaire; QoL-AD Quality of Life in Alzheimer’s Disease; SwLS Satisfaction with Life Scale; WHO-5 World Health Organization-Five Well-Being Index. Categorical outcome variables are presented as percentages (SE), and continuous variables as mean (SE). Bold indicates significance at the 5% level after Holm-Bonferroni correction. Where significant, post hoc comparisons between each class were reported if P < 0.005, and the significant differences are denoted by numbered superscripts which correspond with class number or no problem group (NP). For example, under the results for Class 1, superscripts 2, 3 and 4 indicate that the mean for the given variable was significantly different from Classes 2, 3 and 4 after Bonferroni correction.

1. *Scores on study variables for carers*

| Measure | Class 1 | Class 2 | Class 3 | Class 4 | No problem (NP) | Comparison |
| --- | --- | --- | --- | --- | --- | --- |
|  | Mean (SE)  % missing | Mean (SE)  % missing | Mean (SE)  % missing | Mean (SE)  % missing | Mean (SE)  % missing |  |
| RSS stress | 18.13 (1.02)  19.7% | 19.40 (0.56)  25.4% | 19.63 (1.01)  24.8% | 19.60 (0.83)  23.3% | 18.11 (1.21)  25.0% | $\chi^{2}$ (4) = 2.42, P = 0.660 |
| NPI-Q distress | 8.38 (0.87)  39.4% | 7.25 (0.42)  41.5% | 6.30 (0.74)  39.3% | 6.79 (0.60)  45.2% | 6.40 (0.67)  43.4% | $\chi^{2}$ (4) = 4.69, P = 0.321 |
| Role captivity | 5.18 (0.22)  19.8% | 5.54 (0.13)  24.0% | 5.85 (0.25)  24.1% | 5.62 (0.20)  23.3% | 5.40 (0.27)  25.0% | $\chi^{2}$ (4) = 4.71, P = 0.318 |
| Competence | 8.99 (0.20)  19.8% | 9.02 (0.09)  23.4% | 9.04 (0.19)  21.6% | 9.19 (0.14)  23.2% | 9.64 (0.18) ^1,2,3^  23.7% | $\chi^{2}$ (4) = 10.48, P = 0.033 |
| Positive aspects | 28.86 (0.88)  20.4% | 28.29 (0.39)  23.8% | 28.71 (0.91)  22.2% | 27.26 (0.59)  23.3% | 30.46 (0.96)  25.0% | $\chi^{2}$ (4) = 8.66, P = 0.070 |

*Note.* NPI-Q Neuropsychiatric Inventory Questionnaire; RSS Relative Stress Scale

**Table S6**

*Sensitivity analysis for incorporation of MMSE score (15-17, 18-21, 22-25, 26-30) and years since diagnosis as covariates into the univariable model shown in Table 4.*

| Dementia Representation | Model 1. Univariable | Model 2. Univariable adjusted for MMSE and years since diagnosis |
| --- | --- | --- |
| Age  Class 1. Disease - diagnosis  Class 3. Ageing  Class 4. Unclear  No problem | 0.959 (0.941 – 0.978)*  1.107 (1.079 – 1.135)*  1.007 (0.991 – 1.022)  1.040 (1.004 – 1.078)* | 0.954 (0.934 – 0.975)*  1.112 (1.082 – 1.144)*  1.010 (0.994 – 1.027)  1.041 (0.999 – 1.084) |
| Dementia subtype (AD/VaD/Mixed AD/VaD vs Other)  Class 1. Disease - diagnosis  Class 3. Ageing  Class 4. Unclear  No problem | 0.691 (0.441 – 1.083)  1.730 (0.891 – 3.361)  1.065 (0.723 – 1.567)  1.018 (0.502 – 2.066) | 0.641 (0.398 – 1.032)  1.541 (0.779 – 3.049)  1.085 (0.719 – 1.637)  0.957 (0.450 – 2.036) |
| Functional ability (FAQ-I)  Class 1. Disease - diagnosis  Class 3. Ageing  Class 4. Unclear  No problem | 0.986 (0.967 – 1.005)  1.004 (0.980 – 1.029)  1.006 (0.988 – 1.024)  1.036 (0.997 – 1.076) | 1.001 (0.977 – 1.027)  1.010 (0.983 – 1.038)  0.998 (0.977 – 1.019)  1.034 (0.989 – 1.082) |
| Co-morbidity (CCI)  Class 1. Disease - diagnosis  Class 3. Ageing  Class 4. Unclear  No problem | 1.073 (0.981 – 1.174)  1.108 (1.034 – 1.186)*  1.025 (0.963 – 1.091)  1.031 (0.916 – 1.161) | 1.074 (0.967 – 1.194)  1.120 (1.037 – 1.210)*  1.041 (0.975 – 1.111)  1.064 (0.922 – 1.227) |
| Attitudes towards own ageing  Class 1. Disease - diagnosis  Class 3. Ageing  Class 4. Unclear  No problem | 0.878 (0.785 – 0.983)*  1.268 (1.128 – 1.398)*  1.205 (1.109 – 1.309)*  1.788 (1.509 – 2.118)* | 0.887 (0.788 – 0.998)*  1.300 (1.147 – 1.474)*  1.198 (1.097 – 1.310)*  1.630 (1.366 – 1.945)* |
| Self-efficacy  Class 1. Disease - diagnosis  Class 3. Ageing  Class 4. Unclear  No problem | 0.957 (0.925 – 0.990)*  1.059 (1.023 – 1.091)*  1.043 (1.019 – 1.065)*  1.108 (1.046 – 1.163)* | 0.957 (0.921 – 0.994)*  1.067 (1.026 – 1.110)*  1.048 (1.022 – 1.075)*  1.100 (1.034 – 1.170)* |
| Depression (GDS-10)  Class 1. Disease - diagnosis  Class 3. Ageing  Class 4. Unclear  No problem | 1.127 (1.053 – 1.206)*  0.849 (0.779 – 0.926)*  0.907 (0.854 – 0.963)*  0.632 (0.517 – 0.772)* | 1.100 (1.021 – 1.185)*  0.836 (0.762 – 0.917)*  0.897 (0.843 – 0.956)*  0.654 (0.524 – 0.816)* |

*Note.* AD Alzheimer’s disease; VaD vascular dementia; CCI Charlson Comorbidity Index; FAQ-I Functional Activities Questionnaire-Informant rating; GDS-10 Geriatric Depression Scale 10-items; MMSE Mini-Mental State Examination. * P < 0.05.

**Table S7**

*Comparison of class composition between the full sample and the reduced sample that would have been utilized in calculating the slope (those with 2 or more time points) in the latent growth models shown in Table 5.*

| Domain | Class 1 Disease – diagnosis | | | | Class 2 Disease – symptoms | | | | Class 3 Ageing | | | | Class 4 Unclear | | | |
| --- | --- | --- | --- | --- | --- | --- | --- | --- | --- | --- | --- | --- | --- | --- | --- | --- |
|  | Total (%) | QoL-AD  (%) | SwLS  (%) | WHO-5  (%) | Total  (%) | QoL-AD  (%) | SwLS  (%) | WHO-5  (%) | Total  (%) | QoL-AD  (%) | SwLS  (%) | WHO-5  (%) | Total  (%) | QoL-AD  (%) | SwLS  (%) | WHO-5  (%) |
| IDENTITY |  |  |  |  |  |  |  |  |  |  |  |  |  |  |  |  |
| Diagnostic label | 43.9 | 40.6 | 43.0 | 41.6 | 29.0 | 29.9 | 30.4 | 30.8 | 10.5 | 14.1 | 13.2 | 13.4 | 25.9 | 25.0 | 24.4 | 24.2 |
| Descriptive - symptoms | 31.1 | 34.3 | 32.1 | 32.4 | 48.4 | 46.7 | 46.1 | 45.9 | 57.1 | 59.7 | 61.4 | 60.6 | 46.5 | 47.3 | 48.6 | 49.2 |
| Descriptive - emotional | 11.7 | 12.3 | 13.2 | 13.1 | 5.6 | 5.5 | 6.1 | 6.1 | 0.0 | 0.0 | 0.0 | 0.0 | 5.1 | 5.1 | 4.9 | 4.6 |
| Don’t know | 4.6 | 4.0 | 3.6 | 4.6 | 10.1 | 10.1 | 9.5 | 9.4 | 9.1 | 7.7 | 5.7 | 7.6 | 14.6 | 14.3 | 14.1 | 13.5 |
| Other | 8.7 | 8.8 | 8.0 | 8.3 | 6.8 | 7.8 | 8.0 | 7.8 | 23.3 | 18.5 | 19.7 | 18.4 | 7.8 | 8.4 | 8.0 | 8.4 |
|  |  |  |  |  |  |  |  |  |  |  |  |  |  |  |  |  |
| CAUSE |  |  |  |  |  |  |  |  |  |  |  |  |  |  |  |  |
| Ageing | 4.4 | 3.2 | 2.9 | 3.1 | 21.3 | 19.7 | 19.7 | 19.8 | 100 | 100 | 100 | 100 | 0.0 | 0.0 | 0.0 | 0.0 |
| Brain/disease/  hereditary | 57.2 | 57.3 | 57.8 | 57.3 | 44.7 | 45.9 | 46.2 | 46.1 | 0.0 | 0.0 | 0.0 | 0.0 | 45.0 | 48.1 | 48.4 | 47.9 |
| Lifestyle/life events | 12.9 | 14.2 | 14.7 | 13.8 | 9.2 | 10.0 | 9.9 | 9.7 | 0.0 | 0.0 | 0.0 | 0.0 | 13.1 | 15.4 | 14.6 | 14.9 |
| Don’t know/  unclassifiable | 25.4 | 25.2 | 24.6 | 25.8 | 24.8 | 24.4 | 24.1 | 24.3 | 0.0 | 0.0 | 0.0 | 0.0 | 41.8 | 36.5 | 37.0 | 37.2 |
|  |  |  |  |  |  |  |  |  |  |  |  |  |  |  |  |  |
| TIMELINE |  |  |  |  |  |  |  |  |  |  |  |  |  |  |  |  |
| Better | 0.9 | 1.0 | 0.9 | 0.9 | 0.2 | 0.2 | 0.3 | 0.3 | 10.8 | 10.0 | 9.4 | 10.7 | 16.8 | 16.7 | 17.2 | 18.0 |
| Same | 3.4 | 4.0 | 3.6 | 3.6 | 1.2 | 1.3 | 1.3 | 1.4 | 43.9 | 46.5 | 45.9 | 44.3 | 37.6 | 40.5 | 39.6 | 39.9 |
| Worse | 75.8 | 75.6 | 77.3 | 77.9 | 68.2 | 66.5 | 66.4 | 66.3 | 2.6 | 1.9 | 2.2 | 2.1 | 3.3 | 3.5 | 3.4 | 3.4 |
| Unsure | 19.9 | 19.4 | 18.3 | 17.6 | 30.4 | 32.0 | 32.0 | 32.1 | 42.7 | 41.6 | 42.5 | 42.9 | 42.3 | 39.4 | 39.8 | 38.8 |
|  |  |  |  |  |  |  |  |  |  |  |  |  |  |  |  |  |
| CONTROL  Strongly agree  Agree  Disagree  Strongly disagree | 10.5  41.2  33.5  14.9 | 10.1  39.6  36.4  13.8 | 9.1  41.1  34.4  15.3 | 10.1  40.6  34.7  14.5 | 4.4  61.1  32.7  1.8 | 4.6  63.4  30.3  1.7 | 4.4  64.1  30.0  1.6 | 4.3  63.8  30.3  1.6 | 7.1  63.0  29.9  0.0 | 6.6  65.5  27.9  0.0 | 5.9  66.2  27.9  0.0 | 6.3  65.6  28.1  0.0 | 9.0  61.3  27.3  2.4 | 8.6  64.3  25.0  2.2 | 8.9  64.3  24.2  2.5 | 8.8  64.1  24.6  2.5 |

*Note.* QoL-AD Quality of Life in Alzheimer’s Disease, SwLS Satisfaction with Life Scale, WHO-5 World Health Organization-Five Well-Being Index

**Figure S1**

*Latent growth curve model for quality of life at Times 1-3 (T1-T3).* *The model was replicated for satisfaction with life and well-being.*


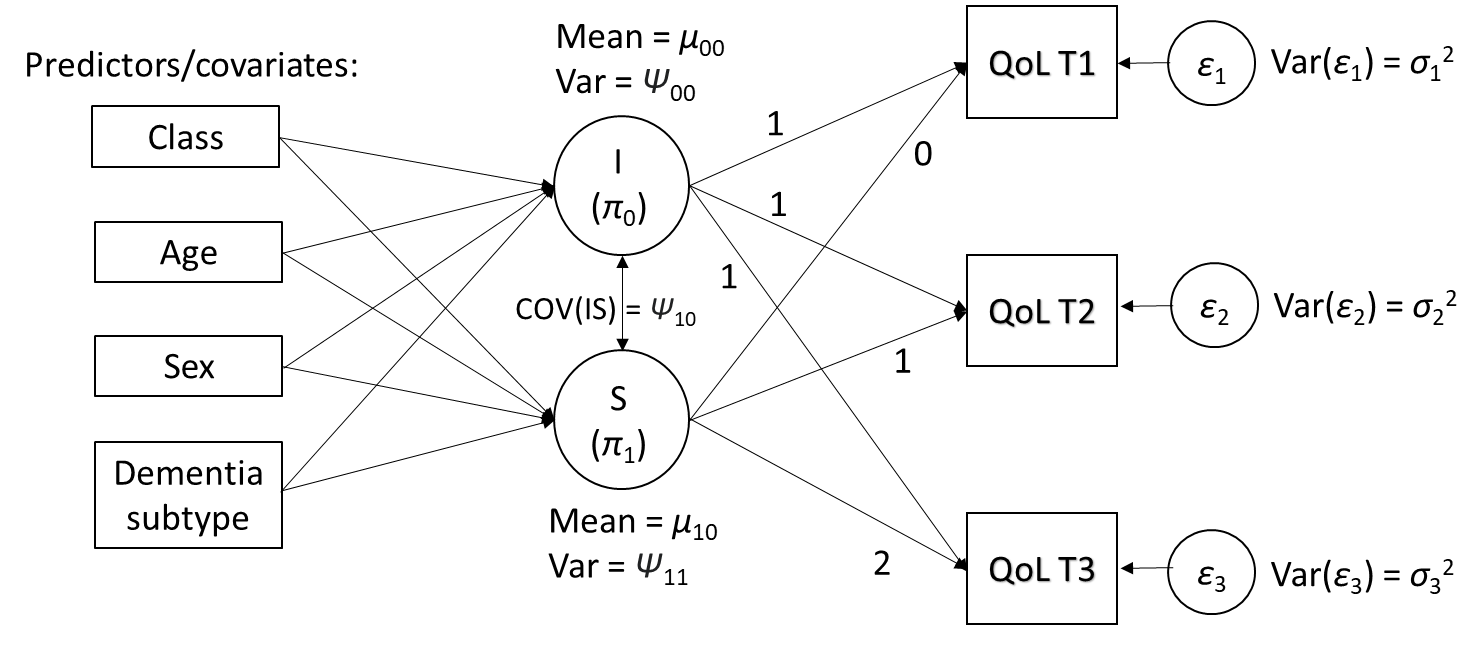


*Note.* The intercept (I) and slope (S) are measured by the three manifest variables QoL T1, QoL T2 and QoL T3. The coefficients of π_0_ represent the factor loadings of the 3 measurements at baseline and are set to 1. The coefficients of π_1_ correspond to the factor loadings of the three measurement occasions for the slope and are set to 0, 1, and 2 to represent the equidistant yearly spacing of the three waves.

**Figure S2**

*Problem-focused coping as a mediator of the relationship between class membership and quality of life score.* *Problem focused coping at Time 2 and quality of life at Time 3 are used in this model.* *The model was replicated for satisfaction with life and well-being.*

For the quality of life model, only 408 participants had data for all three measurements (T1 class, T2 problem-focused coping, T3 QoL-AD). For the satisfaction with life model, only 443 participants had data for all three measurements (T1 class, T2 problem-focused coping, T3 SwLS). For well-being, only 450 participants had data for all three measurements (T1 class, T2 problem-focused coping, T3 WHO-5).


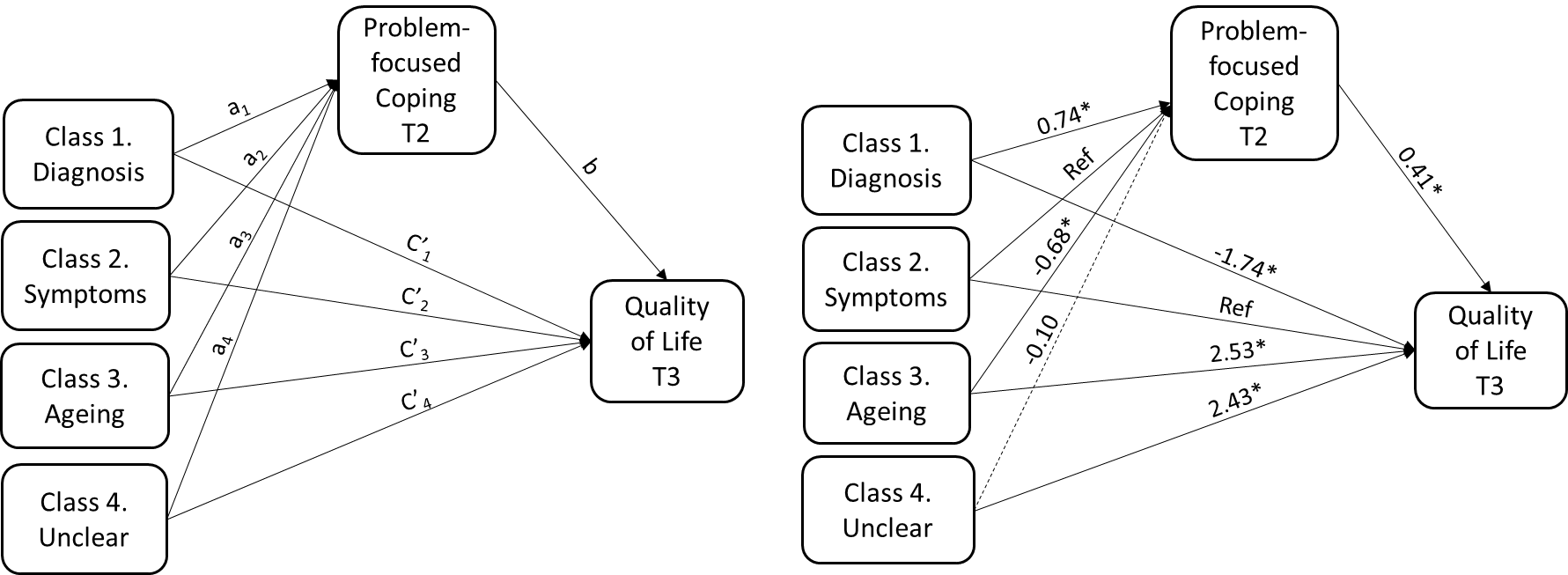


Summary of direct and indirect effects relative to class 2 (disease-symptoms) for quality of life, satisfaction with life and well-being outcome measures

|  | **Relative indirect effect** | **Relative direct effect** |
| --- | --- | --- |
| *Quality of Life* | | |
| Class 1 Disease - diagnosis | 0.31 (0.07 – 0.77) | -1.74 (-3.44 - -0.12)* |
| Class 2 Disease - symptoms | Ref. | Ref. |
| Class 3 Ageing | -0.28 (-0.71 – -0.04)* | 2.53 (0.60 – 3.97)* |
| Class 4 Unclear | -0.04 (-0.24 – 0.12) | 2.43 (0.92 – 3.73)* |
| *Satisfaction with Life* | | |
| Class 1 Disease - diagnosis | 0.20 (0.00 – 0.59) | -2.90 (-5.01– -0.75)* |
| Class 2 Disease - symptoms | Ref. | Ref. |
| Class 3 Ageing | -0.19 (-0.59 – -0.01)* | 3.15 (1.47 – 4.56)* |
| Class 4 Unclear | -0.03 (-0.21 – 0.07) | 1.58 (0.06 – 3.05)* |
| *Well-being* | | |
| Class 1 Disease - diagnosis | 1.08 (0.22 – 2.72)* | -9.40 (-16.90 – -3.41)* |
| Class 2 Disease - symptoms | Ref. | Ref. |
| Class 3 Ageing | -1.02 (-2.68 – -0.20)* | 8.01 (2.27 – 14.19)* |
| Class 4 Unclear | -0.14 (-0.74 – 0.49) | 5.94 (0.26 – 10.94)* |

*Note*. * P < 0.05

Estimates for the pathway between problem-focused coping and each living well measure (path *b*):

Problem-focused coping -> quality of life = 0.41 (95% CI 0.15 – 0.69).

Problem-focused coping -> satisfaction with life = 0.28 (95% CI 0.02 – 0.53).

Problem-focused coping -> well-being = 1.50 (95% CI 0.59 – 2.39).
